# Arm Based on LEg blood pressures (ABLE-BP): can systolic ankle blood pressure measurements predict systolic arm blood pressure? An individual participant data meta-analysis from the INTERPRESS-IPD Collaboration

**DOI:** 10.1136/bmjopen-2024-094389

**Published:** 2025-06-11

**Authors:** Sinead T J McDonagh, Fiona C Warren, James Peter Sheppard, Kate Boddy, Leon Farmer, Helen Shore, Phil Williams, Philip S Lewis, A Jayne Fordham, Una Martin, Victor Aboyans, Christopher Elles Clark, on behalf of the INTERPRESS-IPD Collaborators, James White

**Affiliations:** 1 Health & Community Sciences, University of Exeter Medical School, Exeter, UK; 2 Nuffield Department of Primary Care Health Sciences, University of Oxford Division of Public Health and Primary Health Care, Oxford, UK; 3 Institute for Health Research, University of Exeter Medical School, Exeter, UK; 4 Patient and Public Advisor, Exeter, UK; 5 Cardiology, Stepping Hill Hospital, Stockport, UK; 6 Mid Devon Medical Practice, Tiverton, UK; 7 University of Birmingham College of Medical and Dental Sciences, Birmingham, UK; 8 Department of Cardiology, Dupuytren University Hospital, and Inserm 1094, Tropical Neuroepidemiology, Limoges, France

**Keywords:** Hypertension, Primary Health Care, Cardiology

## Abstract

**Abstract:**

**Objectives:**

To determine associations between arm and ankle systolic blood pressures (SBPs), develop and validate a multivariable model predicting arm SBP from ankle SBP, and investigate associations between ankle SBP, cardiovascular disease and mortality.

**Design:**

Ankle-arm SBP differences were examined in two-stage individual participant data (IPD) meta-analyses using multivariable hierarchical linear regression models. Models were used to derive and validate a prediction model for arm SBP based on ankle SBP. Model performance was assessed using area under the receiver operating characteristic (AUROC) curve analyses. Prognostic associations of ankle SBP with outcomes were examined using Cox proportional hazards models.

**Data sources:**

Searches identified cohorts for the Inter-arm Blood Pressure Difference IPD (INTERPRESS-IPD) Collaboration from Medline, Old Medline, Medline in process, Embase and CINAHL databases from inception until January 2017; unpublished data were also sought. Required primary outcomes were all-cause mortality, cardiovascular mortality, and/or fatal and non-fatal cardiovascular events.

**Eligibility criteria:**

Prospective studies from community, primary care or general clinic settings, without language restriction, that recorded SBP in both arms were eligible. Adults aged ≥18 years with SBP measured in all four limbs, in a supine position, were included in the current analyses. People with peripheral artery disease were excluded.

**Data extraction and synthesis:**

Anonymised datasets were individually cleaned and then combined into a single dataset for the INTERPRESS-IPD Collaboration.

**Results:**

The current dataset included 33 710 participants from 14 studies; mean age 58 years, 45% female, mean baseline arm blood pressure 138/80 (SD: 20/12) mm Hg. Mean ankle SBP was 12.0 mm Hg (95% CI 8.8 to 15.2) higher than arm SBP. The multivariable model predicting arm SBP from ankle SBP demonstrated excellent performance (AUROC curves, sensitivities and specificities were >0.82, 0.80 and 0.82, respectively, at all BP thresholds from 130 to 160 mm Hg). Model performance was superior to existing arithmetic formulae.

Ankle SBP was neither associated with all-cause nor cardiovascular mortality (HR 1.000 (0.997 to 1.002; p=0.682) and 1.001 (0.996 to 1.005; p=0.840), respectively). However, lower-reading ankle SBP was associated with fatal or non-fatal cardiovascular events (HR 1.005 (1.002 to 1.007; p<0.001).

**Conclusions:**

On average, ankle SBP is 12 mm Hg higher than arm SBP. Estimating individual arm SBP from ankle SBP measurements with a multivariable model is more accurate than existing fixed arithmetic formulae. This model, operationalised in an online calculator (https://ablebp.research.exeter.ac.uk/), could facilitate hypertension management and cardiovascular care for people unable to have arm SBP measured.

**PROSPERO registration number:**

CRD42015031227.

**STRENGTHS AND LIMITATIONS OF THIS STUDY**

- This is the largest study to date examining the relationship between arm and ankle blood pressure (BP) using individual participant data (IPD) meta-analyses.
- We have created a novel model, using IPD meta-analysis methods, to estimate arm systolic BP from ankle systolic BP; this approach proved superior to existing estimation methods.
- Our prediction model is freely available for use within a web-based calculator online (https://ablebp.research.exeter.ac.uk/).
- IPD meta-analysis can overcome limitations associated with study-level aggregate meta-analyses; however, in this study, heterogeneity still existed and was not explained by sensitivity analyses, so generalisation of results should be undertaken with caution.
- Despite using an international dataset with over 33 000 individuals, further validation of our models in ethnicities and specialised populations (particularly those with limb deformity or loss) not currently represented is required.

## Introduction

 Blood pressure (BP) is, by convention and for convenience, primarily measured on the upper arm; international hypertension guidelines rely entirely on such measurements.^1^,^3^ However, arm (brachial) BP measurement is not always feasible. Approximately 13 people per 100 000 population have upper limb prostheses in the UK, and over 1700 amputations above wrist level occur each year.^4^ Arm BP measurement may not be possible temporarily following multiple fractures, or permanently due to an upper arm amputation, morbid obesity, bilateral lymphoedema, phocomelia, or altered muscle tone and hemiplegia following stroke.^5^,^7^ BP measurement may also be unreliable in the presence of bilateral arterial stenoses due to atheroma or arteritides.^8^ People living with phocomelia or amputation carry additional cardiovascular risks compared with the general population, emphasising their need for careful management of risk factors such as high BP.^7 9^ When arm measurement is not feasible, leg (ankle) BP measurement is recommended as an alternative.^10^ Multiple conversion guidelines exist, creating uncertainty over how to best interpret ankle BP measurements; both subtraction and multiplication conversions to equivalent arm pressures have been proposed; these all take a ‘one size fits all’ approach, assuming that the ankle-arm BP relationship is constant (box 1).^7 10 11^

Box 1 **Current conversion methods for ankle to arm blood pressures**

Existing proposals for adjusting ankle blood pressure (BP) measurements to equivalent brachial blood pressure values:

- Multiply (ankle systolic BP+8)×0.88 (Shiga *et al*^7^).
- Deduct 10/5 mm Hg from ankle systolic/diastolic BP (Thalidomide Trust).
- Deduct 15 mm Hg from ankle systolic; diastolic is unchanged (British and Irish Hypertension Society).

Reported differences between ankle and arm systolic BPs (SBPs), defined as ankle minus arm BP, range from 10 to 40 mm Hg in healthy participants.^12 13^ Our previous study-level systematic review and meta-analysis of 9771 participants from 44 general population studies reported a pooled mean ankle-arm difference in SBP of 17.0 mm Hg (95% CI 15.4 to 21.3 mm Hg); diastolic BP (DBP) difference was −0.3 mm Hg (95% CI −1.5 to 1.0 mm Hg).^14^ Consequently, we concluded that an ankle BP of 155/90 mm Hg could be broadly equated to the current National Institute for Health and Care Excellence (NICE) arm threshold of 140/90 mm Hg.^2^

Within that review, we found substantial residual heterogeneity between studies, suggesting that a single conversion factor may not have equal utility across the full range of participants’ likely ankle BPs and characteristics. We hypothesised that an alternative multivariable modelling approach might improve the accuracy of individual arm BP estimation. The Inter-arm BP Difference Individual Participant Data (INTERPRESS-IPD) Collaboration includes over 33 000 participants with bilateral arm and ankle BP data.^15^ We used these data to establish the Arm Based on LEg BP (ABLE-BP) cohort, to overcome some limitations of study-level meta-analysis and: (1) examine the association between arm and ankle SBP, (2) derive and validate a model to estimate arm SBP based on ankle SBP and (3) explore the association between ankle SBP and cardiovascular events, cardiovascular mortality and all-cause mortality.

## Methods

This IPD meta-analysis was registered with PROSPERO (CRD42015031227) and the protocol has been published.^16^ The study was conducted in accordance with the Preferred Reporting Items for a Systematic Review and Meta-analysis of Individual Participant Data statement.^17^

### Literature search and study identification

Establishment of the INTERPRESS-IPD Collaboration and of the ABLE-BP cohort have been previously described.^16 18^ In brief, we searched Medline, Old Medline, Medline in process, Embase and CINAHL from inception until January 2017 for eligible observational longitudinal studies or randomised controlled trials without BP lowering interventions. Required primary outcomes were all-cause mortality, cardiovascular mortality, and/or fatal and non-fatal cardiovascular events. We contacted the authors of studies identified as likely to hold published or unpublished data on participants with BP recorded in both arms to request access. The subset of our included studies that measured Ankle-Brachial Index (ABI), thus offering supine SBP measurements in four limbs, was eligible for inclusion in the ABLE-BP cohort. Initial data sharing agreements with study lead authors were extended to permit the current analyses. Participants were recruited from primary, community or general care settings and were aged ≥18 years. Participant and study-level characteristics known to relate to cardiovascular risk, including age, sex, ethnicity, body mass index (BMI), smoking status, total and high-density lipoprotein (HDL) cholesterol, pre-existing cardiovascular diagnoses and methods of BP measurement were sought.

### BP measurement

BP measurements were obtained sequentially from all four limbs according to ABI protocols lying supine following a rest period. If more than one sequence was recorded, values from the first sequence were used.^15^ Online supplemental table S4 details BP measurement methods and study-level characteristics.

### Data preparation

Eligible participants in the INTERPRESS-IPD dataset were combined into the new ABLE-BP cohort using Stata V.17.0 (StataCorp, Texas, USA). One potentially eligible study only recruited participants with an ABI≤0.95 and was excluded.^19^ Established peripheral artery disease (PAD) profoundly alters the relationship of leg to arm BP. Therefore, individual participants were excluded if they had PAD, an ABI<0.90, or lower reading ankle SBP<70 mm Hg or >250 mm Hg, such readings being diagnostic or highly indicative of PAD.^20^

### Statistical analysis

DBP is not routinely recorded when measuring ABI and was not available in the INTERPRESS-IPD data, so all analyses examined SBP. We defined the difference between arm and ankle BP as the lower-reading posterior tibial artery SBP minus the higher-reading arm SBP using the first set of four-limb BP readings. Participant characteristics were described at study level, including age, sex, ethnic group, BMI, arm and ankle BP, smoking status, total and HDL cholesterol, and the presence of diabetes mellitus, hypertension, cerebrovascular disease or ischaemic heart disease.

The co-primary outcomes examined were: (1) the ankle-arm SBP difference and (2) arm SBP estimated from ankle SBP using multivariable modelling. We also examined: (3) the association between ankle SBP and all-cause mortality, cardiovascular mortality and non-fatal cardiovascular events (defined as first incidence of myocardial infarction, physician confirmed angina, coronary revascularisation, transient ischaemic attack or stroke). Where an association was detected, we examined the discriminative ability of existing cardiovascular disease (CVD) prediction models (Framingham and AtheroSclerotic CardioVascular Disease (ASCVD) 10-year risk scores) using estimated arm SBP.

For the primary outcomes, multivariable models were developed using observed data only, restricted to participants with no missing data for any model variables (termed ‘complete cases’). Heterogeneity was assessed using the I^2^ and tau^2^ statistics.^21 22^

#### Arm and ankle BP difference

Ankle-arm SBP differences (using higher arm SBP) were reported for each study in two-stage meta-analyses to derive forest plots and assess heterogeneity. Study-level estimates of ankle-arm differences were adjusted for age, sex, BMI, ethnicity, higher-reading arm SBP, smoking status, total cholesterol, pre-existing hypertension, diabetes and CVDs prior to being pooled across studies. Candidate covariates for models were selected for known associations with outcomes and high availability within the INTERPRESS dataset. The association between baseline covariates and ankle-arm SBP was explored in univariable and multivariable models using hierarchical linear regression with a random effect for study.

#### Estimating arm BP using ankle BP

One-stage (comprising hierarchical linear regression with a random effect for study) and two-stage meta-analyses were used to assess heterogeneity using complete case data for the candidate variables of age, sex, BMI, ethnicity, higher-reading arm SBP, smoking status, total cholesterol and medical history. The model was derived using data from a subset of studies (the *derivation* dataset: nine studies, n=21 304) and validated in the remainder (*validation* dataset: four studies, n=8784).^23^ Studies were purposively allocated to the derivation or validation datasets to ensure even distribution of sexes and geographical origin. Model fit was assessed using the Akaike Information Criterion (AIC).^24^ A range of observed higher arm SBP thresholds, from 130 mm Hg to 160 mm Hg in increments of 5 mm Hg, was used to classify participants’ hypertensive status if equal to or greater than each threshold. Using these dichotomised variables, mixed logistic regression models, with a random effect for study, were performed at each threshold, using estimated arm SBP from the derivation cohort model as the predictor in each fitted model. This allowed prediction of the probability of being classed as hypertensive for that threshold, for both the derivation and validation cohorts. A series of area under the receiver operating characteristic (AUROC) curve analyses were used to examine model discrimination for the prediction of hypertensive status at each threshold in the derivation and validation datasets.^25^ Furthermore, the optimal cut-point (on the probability scale) for the diagnosis of hypertension at each BP threshold (using a range of thresholds at 5 mm Hg increments from 130 mm Hg to 160 mm Hg) was estimated, along with sensitivity, specificity and AUROC at the estimated optimal cut-point; 95% CIs were derived by bootstrap (100 replications with replacement). Receiver operating characteristic curves were also used to compare the final estimation model to current arithmetic conversion methods for arm SBP from ankle SBP (box 1).

Model calibration was assessed using calibration plots, and by estimating the calibration slope (based on a mixed linear model regressing observed higher-reading arm SBP on estimated arm SBP (a coefficient of 1 for the regression slope being ideal) and calibration in the large (intercept from same model; intercept of 0 being ideal). Using the derivation dataset, we performed internal-external cross validation (IECV) for the calibration slope by leaving out each study in turn and fitting the final model using only the remaining studies.^26^ For each excluded study in turn, we then regressed the observed higher-reading arm SBP on the estimated arm SBP derived from the model developed using the dataset that excluded that study. The beta coefficients from the regression models were then pooled using a random effects meta-analysis. We used 140 mm Hg and 160 mm Hg, NICE stage 1 and stage 2 hypertension diagnostic thresholds, to evaluate the models’ sensitivities and specificities in diagnosing hypertension.^2^ Due to our exclusion criterion (ankle SBP<70 mm Hg), the prediction of hypotension was outside the scope of our modelling.

#### Association between ankle BP and CVD

For participants without pre-existing CVD, individual time-to-event models were used to examine the association between higher- or lower-reading ankle SBP and all-cause and cardiovascular mortality, and for fatal or non-fatal cardiovascular events. One-stage random effects flexible parametric models failed to converge. Therefore, fixed effect one-stage Cox proportional hazards models were used, stratified by study, with ankle SBP as the main exposure, adjusted for the covariates described above.

Finally, the effects of including model estimated arm SBP (from aim 2) in existing cardiovascular risk prediction models were examined. For this, we used the ASCVD and Framingham 10-year cardiovascular risk scores, comparing scores calculated using the model-estimated arm SBP with scores using observed arm SBP.^27 28^ Analyses were restricted to valid age ranges for each score (ASCVD: 40–79, Framingham: 20–79 years).

#### Sensitivity analyses

In sensitivity analyses, each of the analyses described above was repeated following multiple imputation using chained equations of missing baseline data for participant characteristics and BP variables. All imputation models included participant characteristics and accounted for the study. Imputation models for baseline predictor variables to be included in time-to-event modelling included the event indicator and Nelson-Aalen estimate of the cumulative hazard derived from observed data.^29^ Missing outcome data for time-to-event models was not imputed. Imputation for models predictive of ankle-arm difference and higher-reading arm BP included the outcome variable within the imputation model. Planned sensitivity analyses examining height as a variable in the final models were not feasible due to the global absence of height data. Exploratory back calculation of missing ankle SBP from ABI records proved unreliable when compared with known ankle data; therefore, this was not pursued.

### Patient and public involvement

Three public advisors with lived experience of barriers to measuring arm BP were fully integrated into the project management team, facilitated by KB. Advisors attended pre-meetings and project meetings, contributing to all project stages from protocol development through to dissemination of findings. Their contributions included aligning the project objectives with outcomes meaningful to patients, ensuring that patient and public views were represented, and that results were summarised in accessible formats.^16^

## Results

Fourteen studies contributed data to the ABLE-BP dataset (online supplemental figure S1).^30^,^43^ Included studies originated from Europe (n=7), USA (n=6) and Sub-Saharan Africa (n=1); their details and inclusion criteria have been previously described (online supplemental tables S1–S4).^16^ Records for 33 710 participants contained at least one set of arm and ankle SBP readings. Mean age was 58 years (SD: 13, range: 18–99), 45% were female and baseline mean BP was 138/80 (SD: 20/12) mm Hg; participant characteristics are summarised in table 1.

**Table 1:** Characteristics of participants from 14 studies included in the Arm Based on LEg blood pressure cohort

	N	Mean (SD)
Age (years)	33 710	58.4 (13.3)
Higher arm systolic blood pressure (mm Hg)	33 694	137.7 (20.3)
Diastolic blood pressure (mm Hg)	25 167	79.7 (12.1)
Lower leg SBP	33 710	151.6 (23.4)
Body mass index (kg/m^2^)	33 603	26.9 (5.2)
Total cholesterol (mmol/L)	33 316	5.3 (1.2)
HDL cholesterol (mmol/L)	31 395	1.3 (0.4)
	**N**	**n (%)**
Female	33 710	15 062 (44.7)
Current smoker	33 710	6785 (20.1)
Hypertension	33 710	20 191 (59.9)
Diabetes mellitus	33 710	4917 (14.6)
Cardiovascular disease	33 710	5797 (17.2)
Ischaemic heart disease	32 391	5474 (16.9)
Cerebrovascular disease	33 663	1900 (5.6)
Ethnicity	33 710	
White	24 690 (77.5)	
African American	2880 (9.0)	
Hispanic American	1918 (6.0)	
Black African	679 (2.2)	
Other	1680 (5.3)	

HDL, high-density lipoprotein; SBP, systolic blood pressure.

### Ankle-arm SBP difference

**Figure 1:**
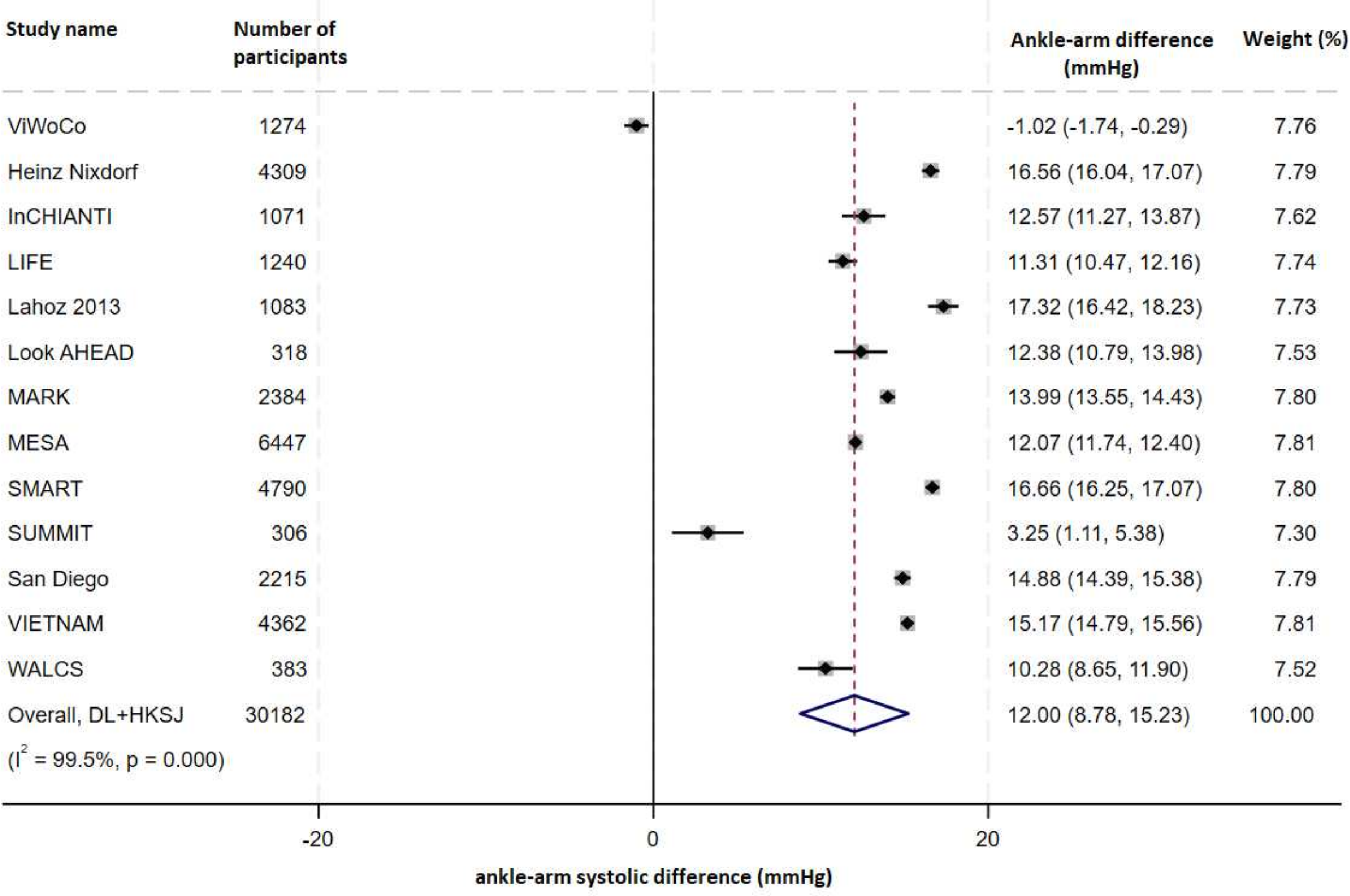
Forest plot of ankle-arm systolic blood pressure (SBP) difference using higher-reading arm SBP.

Using complete case data (n=30 182), two-stage random effects modelling found the mean ankle-arm SBP difference was 12.0 (95% CI 8.8 to 15.2) mm Hg. Heterogeneity was significant (I^2^=99.5%, tau^2^=16.7, p<0.001; figure 1) and did not reduce substantially after adjustment for covariates (I^2^=97.9%, tau^2^=0.01, p<0.001).

Higher-reading arm SBP, female sex, smoking, total cholesterol, ischaemic heart disease and being of African American descent (vs ‘white’ reference) were associated with smaller ankle-arm SBP differences (p<0.05; table 2). Age, BMI, hypertension and being of Hispanic American descent (vs ‘white’ ethnicity reference) were associated with increased ankle-arm SBP differences (p<0.05).

**Table 2:** Multivariable associations between participant characteristics and the difference between lower ankle and higher arm systolic blood pressure

Ankle-arm SBP difference	Coefficient	95% CI	P value
Higher-reading arm SBP (mm Hg)	−0.149	−0.158 to −0.139	<0.001
Age (years)	0.021	0.002 to 0.0401	0.030
Female	−6.147	−6.510 to −5.784	<0.001
Smoker	−3.526	−3.945 to −3.108	<0.001
BMI (kg/m^2^)	0.233	0.199 to 0.267	<0.001
Total cholesterol (mmol/L)	−0.278	−0.431 to −0.125	<0.001
Hypertension	0.784	0.402 to 1.165	<0.001
Diabetes mellitus	−0.526	−1.020 to −0.0311	0.037
Ischaemic heart disease	−4.66	−5.334 to −3.987	<0.001
Ethnicity			
African American	−2.31	−2.928 to −1.688	<0.001
Hispanic American	1.425	0.684 to 2.166	<0.001
Other	−0.459	−1.200 to 0.283	0.226
Constant	30.329	27.277 to 33.381	<0.001

BMI, body mass index; SBP, systolic blood pressure.

Sensitivity analyses using multiple imputation to account for missing data gave similar results to the primary complete case analysis (online supplemental table S5).

### Arm BP estimated from ankle BP

Using 2-stage meta-analysis models to estimate higher arm SBP using lower ankle SBP, heterogeneity was found to be high (I^2^=99%), with similar results using higher ankle SBP. Due to the high level of missing data for HDL cholesterol, this predictor was excluded from models using observed data only. The model using higher-reading ankle SBP demonstrated better model fit than that using lower-reading ankle SBP (AICs: 163 292 vs 163 962, respectively). BMI was not a significant predictor of arm SBP but was retained in the final model for face validity (table 3). Mean (SD) observed arm SBP in the derivation dataset was 137 (20) mm Hg; mean estimated arm SBP in the derivation dataset was 138 (16) mm Hg (calibration slope 1.00 (95% CI 0.99 to 1.01), calibration in the large (intercept) 0.00 (−2.85 to 2.85)). Mean (SD) observed arm SBP in the validation dataset was 137 (21) mm Hg (calibration slope 0.96 (95% CI 0.94 to 0.97), calibration in the large 1.05 (−1.9 to 4.0)). Calibration plots showing estimated arm SBP and observed arm SBP are displayed in online supplemental figure S2. Mean (SD) estimated arm SBP in the validation dataset was 142 (17) mm Hg.

**Table 3:** Ankle-arm systolic blood pressure estimation model

	Coefficient	Lower bound of 95% CI	Upper bound of 95% CI	P value
Higher-reading ankle SBP (mm Hg)	0.59	0.58	0.60	<0.001
Age (years)	0.19	0.17	0.21	<0.001
Female	4.28	3.91	4.65	<0.001
Smoker	1.90	1.52	2.28	<0.001
BMI (kg/m^2^)	0.02	−0.02	0.05	0.37
Total cholesterol (mmol/L)	0.78	0.64	0.93	<0.001
Hypertension	6.45	6.08	6.81	<0.001
Diabetes mellitus	0.48	0.02	0.93	0.04
Cerebrovascular disease	1.55	0.82	2.29	<0.001
Ischaemic heart disease	−3.00	−3.69	−2.31	<0.001
Ethnicity				
African American	2.62	2.07	3.18	<0.001
Hispanic American	0.00	−0.68	0.68	1.00
Other	1.04	0.37	1.70	<0.001
Constant	23.81	20.75	26.87	<0.001

BMI, body mass index; SBP, systolic blood pressure.

The overall pooled calibration slope, following IECV, was 0.96 (95% CI 0.85 to 1.07), indicating acceptable performance of the final model produced using the derivation dataset.^26^

**Figure 2:**
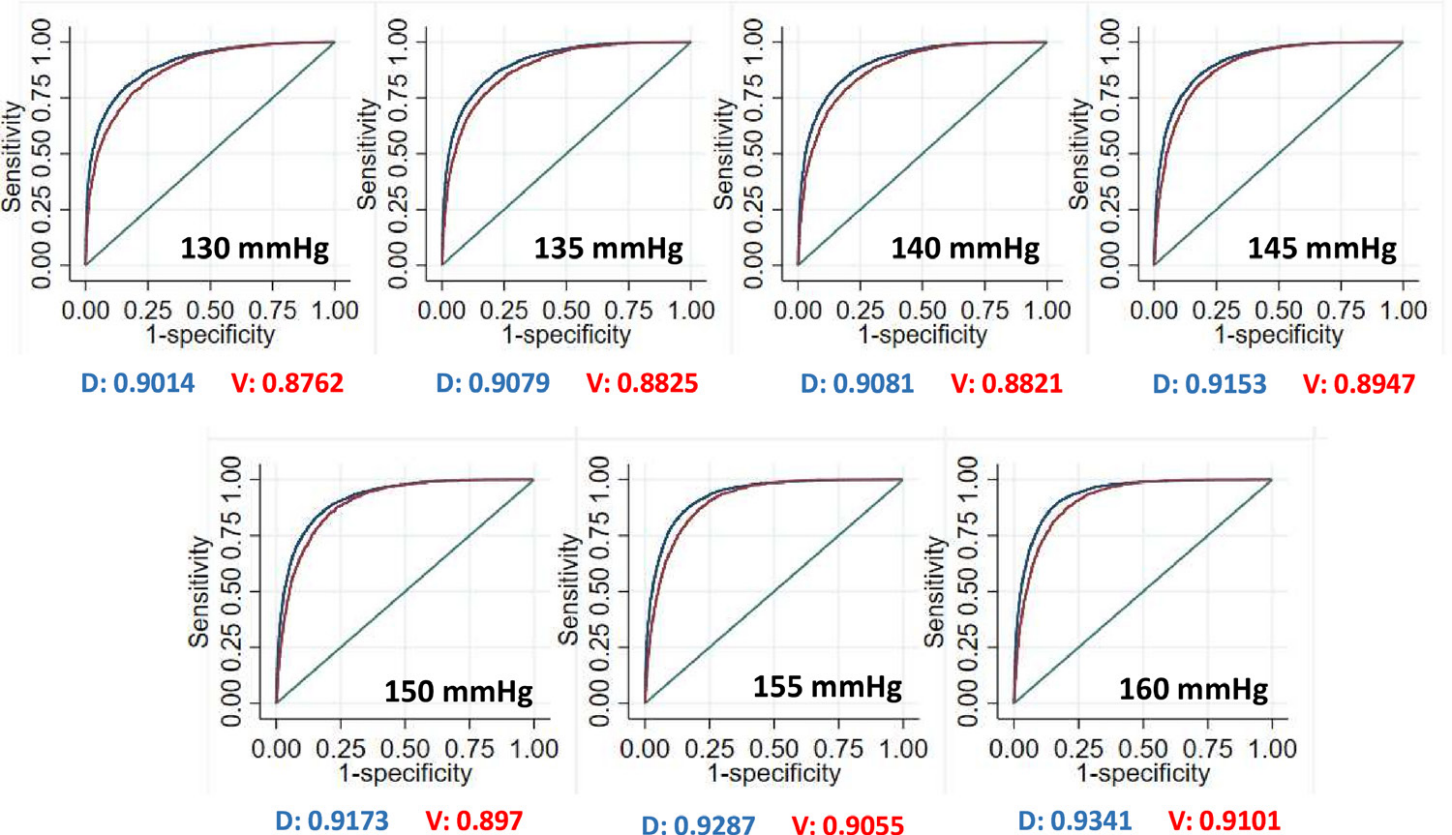
Area under receiver operating characteristic curves for the ankle-arm estimation model for the derivation and validation datasets across blood pressure thresholds from 130 to 160 mm Hg in 5 mm Hg increments. D, derivation cohort; V, validation cohort.

On including HDL as a covariate in the model, including observed and imputed data, HDL was not found to be a significant predictor. In sensitivity analyses, models including observed and imputed data were similar to those using observed data only. Therefore, models using observed data only were retained to assess the performance of the estimation model. We quantified the performance of the ankle-arm estimation model using AUROC curves across a range of BPs (figure 2). AUROC, sensitivity and specificity remained above 0.82, 0.80 and 0.82, respectively, at all thresholds at 5 mm Hg increments from 130 to 160 mm Hg (table 4).

**Table 4:** Arm blood pressure estimated from ankle blood pressure at 5 mm Hg thresholds from 130 to 160 mm Hg

SBP (mm Hg) threshold*	Dataset	OR† (95% CI)	Area under ROC curve‡ (95% CI)	Estimated optimal cutpoint§ (95% CI)	Sensitivity at estimated optimal cutpoint (95% CI)	Specificity at estimated optimal cutpoint (95% CI)	Area under ROC curve at estimated optimal cutpoint (95% CI)
160	Derivation	1.17 (1.16 to 1.18)	0.93 (0.93 to 0.94)	0.15 (0.12 to 0.18)	0.90 (0.87 to 0.92)	0.83 (0.81 to 0.86)	0.86 (0.86 to 0.87)
	Validation	1.13 (1.13 to 1.14)	0.91 (0.90 to 0.92)	0.16 (0.13 to 0.19)	0.88 (0.84 to 0.91)	0.80 (0.76 to 0.83)	0.84 (0.83 to 0.84)
155	Derivation	1.17 (1.16 to 1.18)	0.93 (0.92 to 0.93)	0.23 (0.20 to 0.26)	0.86 (0.83 to 0.88)	0.85 (0.83 to 0.87)	0.85 (0.85 to 0.86)
	Validation	1.13 (1.13 to 1.14)	0.91 (0.90 to 0.91)	0.19 (0.16 to 0.22)	0.86 (0.83 to 0.90)	0.79 (0.76 to 0.83)	0.83 (0.82 to 0.84)
150	Derivation	1.17 (1.16 to 1.17)	0.92 (0.91 to 0.92)	0.31 (0.27 to 0.35)	0.83 (0.81 to 0.86)	0.84 (0.82 to 0.87)	0.84 (0.83 to 0.84)
	Validation	1.14 (1.13 to 1.14)	0.90 (0.89 to 0.90)	0.28 (0.25 to 0.30)	0.85 (0.83 to 0.87)	0.79 (0.78 to 0.81)	0.82 (0.81 to 0.83)
145	Derivation	1.17 (1.16 to 1.17)	0.92 (0.91 to 0.92)	0.34 (0.30 to 0.38)	0.85 (0.82 to 0.88)	0.82 (0.79 to 0.84)	0.84 (0.83 to 0.84)
	Validation	1.14 (1.13 to 1.15)	0.89 (0.89 to 0.90)	0.37 (0.31 to 0.43)	0.81 (0.76 to 0.85)	0.82 (0.78 to 0.87)	0.82 (0.81 to 0.82)
140	Derivation	1.17 (1.17 to 1.18)	0.91 (0.90 to 0.91)	0.47 (0.42 to 0.53)	0.82 (0.79 to 0.85)	0.83 (0.80 to 0.86)	0.83 (0.82 to 0.83)
	Validation	1.14 (1.13 to 1.14)	0.88 (0.88 to 0.89)	0.46 (0.43 to 0.50)	0.81 (0.78 to 0.83)	0.79 (0.77 to 0.82)	0.80 (0.79 to 0.81)
135	Derivation	1.18 (1.17 to 1.18)	0.91 (0.90 to 0.91)	0.56 (0.51 to 0.61)	0.82 (0.79 to 0.84)	0.83 (0.81 to 0.86)	0.82 (0.82 to 0.83)
	Validation	1.14 (1.13 to 1.15)	0.88 (0.88 to 0.89)	0.55 (0.49 to 0.60)	0.79 (0.75 to 0.83)	0.80 (0.77 to 0.84)	0.80 (0.79 to 0.81)
130	Derivation	1.18 (1.18 to 1.19)	0.90 (0.90 to 0.91)	0.68 (0.67 to 0.70)	0.80 (0.79 to 0.81)	0.84 (0.83 to 0.85)	0.82 (0.81 to 0.83)
	Validation	1.14 (1.13 to 1.15)	0.88 (0.87 to 0.88)	0.68 (0.63 to 0.73)	0.78 (0.74 to 0.82)	0.81 (0.77 to 0.85)	0.79 (0.78 to 0.80)

* Threshold for classification as hypertensive if observed higher arm SBP is at or above threshold.

† OR for classification as hypertensive vs non-hypertensive for an increase of 1 mm Hg in predicted arm SBP.

‡ Area under overall ROC curve, that is, for all possible cutpoints.

§ Estimated optimal cutpoint (probability scale).

ROC, receiver operating characteristic; SBP, systolic blood pressure.

The three pre-existing methods of arithmetical mapping of higher-reading ankle SBP to arm SBP (box 1) all yielded identical AUROCs of 0.841 (95% CI 0.837 to 0.845) because no reclassification occurred between application of each arithmetic model. These compared unfavourably with an AUROC of 0.868 (95% CI 0.864 to 0.872) for the estimation model for the diagnostic threshold of 140 mm Hg. Respective figures for the 160 mm Hg threshold were 0.855 (95% CI 0.849 to 0.861) compared with 0.880 (95% CI 0.874 to 0.885) (p<0.0001 for both thresholds). At both thresholds, this represented 2% fewer participants being misclassified according to their observed arm SBP by the model in comparison to the previous arithmetic models (online supplemental table S6).

### Time to event analysis

#### All-cause mortality and cardiovascular mortality

For participants without pre-existing CVD, neither lower- nor higher-reading ankle SBP were associated, after adjustment for other predictors, with either all-cause mortality (adjusted HRs 1.00 (95% CI 1.00 to 1.00; p=0.682) and 1.00 (1.00 to 1.00; p=0.796), respectively) or cardiovascular mortality (HRs 1.00 (1.00 to 1.01; p=0.840) and 1.00 (1.00 to 1.01; p=0.297), respectively). Global tests for non-proportional hazards in all-cause mortality analyses indicated some evidence for non-proportional hazards (p=0.019 for lower ankle SBP overall model, p=0.004 for higher ankle SBP model), driven largely by total cholesterol and ethnic group; there was weak evidence of non-proportional hazards for lower and higher ankle SBP (p=0.058 and p=0.074, respectively). Little evidence was found to refute the proportional hazards hypothesis for cardiovascular mortality analyses (lower and higher ankle SBP p=0.216 and p=0.235, respectively). Following sensitivity analysis, including imputation of missing baseline data, lower-reading ankle SBP was predictive of all-cause and cardiovascular mortality (HRs 1.003 (1.001 to 1.005; p=0.005) and 1.007 (1.003 to 1.011; p=0.001), respectively).

#### Fatal and non-fatal cardiovascular events

In a dataset comprising 19 350 observations across 11 studies, lower-reading ankle SBP was significantly associated with fatal and non-fatal cardiovascular events (HR 1.005 (1.002 to 1.007; p<0.001)). Analyses using multiple imputation of missing data gave similar results (HR 1.006 (1.004 to 1.008; p<0.001)). There was strong evidence for non-proportional hazards (global p=0.010), driven by BMI, total cholesterol and hypertension status, with little evidence for non-proportional hazards with lower ankle SBP (p=0.756).

#### Arm BP estimated from ankle BP in existing cardiovascular risk models

Calculated Framingham and ASCVD 10-year risk scores were similar using either observed higher-reading arm SBP or estimated arm SBP in both the derivation cohort (ASCVD: 16.7% (12.7) vs 17.2% (12.6); Framingham: 19.3% (15.1) vs 19.9% (15.1)) and validation cohort (ASCVD: 16.4% (12.6) vs 16.9% (12.7); Framingham: 19.6% (14.4) vs 20.8% (14.3)).

## Discussion

### Principal findings

To our knowledge, this is the first IPD meta-analysis to explore the association between arm and ankle SBP and to offer an individualised estimate of arm SBP based on ankle SBP measurements.

Using one set of supine sequential SBP measurements from ankles and arms, such as are obtained during ABI measurement protocols, we found that ankle SBP was, on average, 12.0 mm Hg (95% CI 8.8 to 15.2) higher than arm SBP. We developed and validated a model to estimate arm BP from ankle BP which demonstrated greater accuracy than current arithmetic methods of conversion. Established 10-year cardiovascular risk scores calculated using model-derived arm SBP appeared equally accurate to those based on directly measured arm SBP, suggesting it could be used to inform cardiovascular risk estimation and to support individual treatment decisions in people unable to have arm BP measured.

### Comparison with other studies

Previous estimates of ankle-arm SBP differences have ranged up to 40 mm Hg.^44^ Our study-level review reported a pooled ankle-arm SBP difference of 17 mm Hg (95% CI 15.4 to 21.3).^14^ That finding underpinned current recommendations of the British and Irish Hypertension Society that equate an ankle BP of 155/90 mm Hg to NICE guideline 140/90 mm Hg BP threshold for hypertension.^2 10^ Other previous recommendations for conversion of ankle BP were based on much smaller sets of data derived from people affected by thalidomide.^7^ The present analysis suggests that the ankle-arm SBP difference may be lower, averaging about 12 mm Hg.

The ankle-arm difference was lower for smokers than non-smokers, in keeping with the preclinical atherosclerotic effects of cigarette consumption observed by others.^45 46^ Sex differences in ABI, with lower values for females than males, frequently persist following multivariable adjustments and may confound the use of a single, non-sex-specific diagnostic threshold for PAD. Such sex differences are reflected in our model, associating female sex with a smaller ankle-arm difference.^47^ Differences are also observed between ethnicities, including in the absence of arterial disease, and likely reflect physiological variation in arterial stiffness and pulse amplification.^20 47^

Since ABI estimation does not require DBP measurement, no ankle DBP data were available, so no ankle-arm DBP models were explored. Our study-level review found no clinically important difference between arm and ankle DBPs.^14^

The ankle-arm estimation model demonstrated excellent performance, with AUROC curves remaining above 0.90 across all SBP thresholds in the derivation cohort and above 0.88 in the validation cohort. In addition, sensitivity and specificity remained above 80% across the SBP thresholds, increasing up to 90% as SBP increased. To our knowledge, this is the first model generated using IPD methods, to estimate supine arm SBP from ankle SBP. Our method proved superior to existing arithmetic estimation methods (summarised in box 1), achieving a 2% lower misclassification rate at relevant SBP thresholds.^7 10 11^ Although this seems small, hypertension is common; the NHS Health Check Programme diagnoses 38 000 new cases annually in England alone.^48^ Therefore, 2% fewer misclassifications represent over 750 potential misdiagnoses per year in England, or tens of thousands globally.

In multivariable models using observed data only, ankle SBP did not predict all-cause or cardiovascular mortality over 10 years. However, after imputation of missing data, the lower-reading ankle SBP was predictive of both outcomes. Prediction of fatal or non-fatal cardiovascular events was consistent across models with or without imputation of missing data for both lower- and higher-reading ankle SBPs. Both low and high ankle BPs have previously been associated with all-cause and cardiovascular mortality.^20 49^ The weaker associations demonstrated here probably reflect our exclusion criteria for both low (indicating PAD) and high (indicating incompressible calcific arteries) ankle SBP values. Overall, cardiovascular risk was predicted equally well using existing cardiovascular risk models with estimated arm SBPs in comparison to observed arm SBPs.

While the higher ankle SBP reading performed better than the lower ankle in estimating arm SBP, the lower-reading ankle performed better in prognostic modelling. These findings were consistent with previous studies and existing consensus recommendations to adopt the higher-reading ankle when determining ABI for the diagnosis of PAD but the lower-reading ankle when using ABI for the prognosis of future events.^20 49^

### Strengths and limitations of the study

Our recent study-level meta-analysis of 9771 participants was, until now, the largest study examining the association between ankle and arm BP.^14^ Conclusions that can be drawn from study-level aggregate meta-analyses are limited since data are combined from different studies with differing population characteristics, analytical approaches and reporting. By using IPD data, we examined the associations between ankle and arm SBP in a considerably larger cohort (over 33 000 participants) than has previously been studied, overcoming some study-level meta-analysis limitations.^15 50^ However, significant residual heterogeneity remained; there were no clear methodological or demographic explanations for individual outlying studies in two-stage analyses, and heterogeneity was not explained using sensitivity analyses, therefore, we exercise some caution in generalising our findings.

These analyses followed an a priori published protocol.^16^ Unlike study-level reviews, the IPD meta-analysis process requires substantial additional time and resources for data cleaning, preparation and analysis, making it less appropriate for reporting state-of-the-art findings, but well suited for more thorough and appropriate modelling and analyses than our previous review.^51^ The INTERPRESS-IPD Collaboration, from which the ABLE-BP dataset has been derived, was established in 2017. The INTERPRESS-IPD search strategy sought cohorts with records of BP from both arms, not all four limbs.^18^ Therefore, recent studies holding potentially eligible data for the current ankle-arm analyses may not have been invited to contribute. However, our inclusion of relevant data for over 33 000 participants in the ABLE-BP dataset has permitted us to model, for the first time, the individual relationships of arm and ankle BPs and to draw robust conclusions from the primary and subgroup analyses.

Our participants were mainly from European and North American cohorts, with one Sub-Saharan Africa cohort. Consequently, further validation of our models for people with ethnicities not currently represented is required. Finally, we necessarily excluded participants with diagnosed or suspected PAD from our analyses. PAD may significantly and unpredictably alter the relationship between ankle and arm SBP, making it inappropriate to apply our models to people with known or suspected PAD. Some participants with sub-clinical PAD, not meeting ABI diagnostic criteria for PAD, will undoubtedly have been represented in the cohorts studied, as would be expected for any sample representative of a wider community. Ankle pressures were predominantly measured using Doppler techniques; other approaches, for example oscillometric ankle pressure measurement, can yield different results, so we do not assume equivalence of our models for different techniques of ankle measurement.^52^ Similarly, any application of our models assumes that ankle measurements were made in the supine position.^53^

Traumatic limb amputation has been associated with subsequent systolic hypertension and increased cardiovascular risk^54 55^; the causes underlying such associations are unclear.^56^ Hypertension and cardiovascular events also appear more common among thalidomide survivors than their matched counterparts.^57^ Our dataset did not include anyone with limb loss; validation of our models in specialised populations with the most to gain from this work, that is, those with limb deformity or loss, would be desirable but challenging to achieve.^7^ We extracted data from community population studies; intercurrent illnesses could not be accounted for, so our findings cannot be applied to acute care or other non-routine settings or populations.

### Implications and conclusions

We conservatively estimate that 6000–10 000 adults may be living with significant congenital or acquired upper limb loss in the UK. Hypertension and CVD are important concerns for people for whom arm BP measurement is not feasible. Current methods for interpreting non-arm BP readings are conflicting, increasing the risk of health inequalities in these individuals. These individuals require accurate interpretation of BP to inform and mitigate their cardiovascular risk and minimise consequences such as stroke.

In addition, there are 1.3 million stroke survivors living in the UK; 75% of whom have upper limb dysfunction impairing activities of daily living.^58^ Self-monitoring and self-titration of BP-lowering treatment achieve lower BPs in people at risk of new or recurrent stroke.^59^ However, arm measurement is difficult or impossible for many stroke survivors who may also, therefore, benefit from ankle BP measurements.

We developed and validated a model that estimates individual arm SBP from ankle SBP measurements using readily available participant characteristics. The higher-reading ankle SBP was a moderately better predictor of higher-reading arm SBP than the lower-reading ankle pressure. To facilitate the use of ankle SBP to estimate arm SBP in individual consultations in patients where arm assessment is not possible or advised, we have developed a freely available, easy-to-use, web-based calculator based on the final model (https://ablebp.research.exeter.ac.uk/).

## Supplementary material

10.1136/bmjopen-2024-094389 online supplemental file 1

## Data Availability

No data are available.
