# Supplementary material for: Arm Based on LEg blood pressures (ABLE-BP): can systolic ankle blood pressure measurements predict systolic arm blood pressure? An individual participant data meta-analysis from the INTERPRESS-IPD Collaboration
Source: BMJ Open. 2025 Jun 11;15(6):e094389. doi: 10.1136/bmjopen-2024-094389 (PMC12161303; doi:10.1136/bmjopen-2024-094389)
Supplement: online supplemental file 1 [file bmjopen-15-6-s001.pdf]

Online Data Supplement

Arm Based on LEg blood pressures (ABLE-BP): Can systolic ankle blood pressure measurements predict systolic arm blood pressure? An individual participant data meta-analysis from the INTERPRESS-IPD Collaboration

Sinéad TJ McDonagh, PhD[1] ORCID ID: 0000-0002-0283-3095, Fiona C Warren, PhD[1] ORCID ID: 0000-0002-3833-0182, James Sheppard, PhD[2] ORCID ID: 0000-0002-4461-8756, Kate Boddy, MSc[1] ORCID ID: 0000-0001-9135-5488, Leon Farmer[3], Helen Shore[3] Phil Williams[3], Philip S Lewis[4] ORCID ID: 0000-0002-7903-390X, A Jayne Fordham, MSc[5], Una Martin[6], Victor Aboyans[7] ORCID ID: 0000-0002-0322-9818, Christopher E Clark, PhD; ORCID ID: 0000-0002-7526-3038.[1]

1. University of Exeter Medical School, St Luke's Campus, Magdalen Road, Exeter, Devon, England EX1 2LU

2. University of Oxford, Woodstock Road, Oxford, England OX2 6GG

3. Volunteer patient and public advisor

4. Stepping Hill Hospital, Stockport NHS Foundation Trust, Stockport, Cheshire SK2 7JE

5. Mid Devon Medical Practice, Witheridge, Devon, England EX16 8EZ

6. University of Birmingham, Birmingham, England B15 2TT

7. Department of Cardiology, Dupuytren University Hospital, Limoges, France

List of supplemental materials

Table S1 – Study level baseline demographic and health characteristics

Table S2 – Distribution of morbidities at baseline

Table S3 – Study level outcomes and attrition, all participants

Table S4 – Characteristics of included studies

Table S5 - Ankle-arm systolic blood pressure estimation model using observed and imputed data (derivation cohort)

Table S6 – Comparison of models' classification of estimated systolic blood pressure to observed systolic blood pressure at A) 140 mmHg and B) 160 mmHg thresholds

Figure S1 - PRISMA flow chart for literature search

Figure S2 – Calibration plots for observed arm systolic blood pressures against model derived expected arm systolic blood pressures for a) derivation and b) validation datasets

References to included studies

Table S1 – Study level baseline demographic and health characteristics

Study	Total participants N	Total participants included in ABLE BP analyses; n/N (%)	Female; n/N (%)	Age (years); mean (SD); median [min, max]	Systolic arm BP (mmHg); mean (SD); median [min, max]	Diastolic arm BP (mmHg); mean (SD); median [min, max]	Current smoker; n/N (%)	BMI[2]; mean (SD), n; median [min, max]	Total cholesterol (mmol/l); mean (SD), n	HDL cholesterol (mmol/l); mean (SD), n
Viborg Women Cohort (ViWoCo)[1]	1440	1302/1440 (90)	1302/1302 (100)	66.3 (5.07); 66 [59, 77]	147.5 (20.4); 146 [100, 237]	80.5 (9.7); 80 [47, 133]	173/1302 (13)	26.2 (5.1) 1276; 25.3 [12.9, 64.5]	5.83 (0.96), 1302	NR
Epidemiology of dementia in Central Africa (EPIDEMCA)[2]	929	697/929 (75)	404/697 (58)	73.1 (6.61); 72 [65, 99]	145.3 (27.1); 140 [85, 240]	83.3 (14.7); 80 [35, 140]	82/694 (12)	21.2 (4.77) 684; 20 [11, 47]	4.1 (0.97), 584	NR
Heinz Nixdorf Recall Study[3]	4736	4391/4736 (93)	2234/4391 (51)	59.3 (7.75); 60 [45, 76]	137.2 (22.0); 134 [82, 251]	NR	995/4386 (23)	27.9 (4.55) 4370; 27 [17, 54]	5.94 (1.01), 4372	1.51 (0.44), 4370
Invecchiare in Chianti (InCHIANTI)[4]	1258	1106/1258 (88)	629/1106 (57)	66.3 (15.62); 70 [21, 93]	145.1 (21.6); 145 [90, 230]	83.0 (9.5); 80 [60, 120)	195/1106 (18)	27.2 (4.11) 1075; 27 [18, 44]	5.57 (1.03), 1102	1.47 (0.38), 1102
Lifestyle Interventions and Independence for Elders (LIFE) study[5]	1603	1336/1603 (83)	889/1336 (67)	78.1 (5.12); 78 [70, 89]	126.7 (17.5); 125 [80, 197]	68.4 (10.1); 68 [40, 106]	27/1332 (2)	30.5 (5.98) 1336; 29.7 [16.3, 57.6]	4.61 (1), 1262	1.58 (0.46), 1262
Lahoz 2013 (Fuencarral Health Center)[6]	1103	1092/1103 (99)	709/1092 (65)	69.3 (5.24); 69 [60, 79]	141.9 (18.2); 140 [95, 220]	NR	107/1092 (10)	29.1 (4.35) 1092; 29 [16, 48]	5.56 (0.94), 1084	1.52 (0.37), 1084
Action for Health in Diabetes (Look AHEAD)[7]	342	321/342 (94)	193/321 (60)	55.8 (6.97); 55 [45, 74]	131.9 (17.3); 132 [61, 196]	71.5 (9.7); 72 [38, 97]	16/320 (5)	36.4 (5.83) 321; 36 [26, 64]	5.11 (0.94), 321	1.08 (0.28), 321

Improving interMediAte RisK management (MARK) study[8]	2490	2385/2490 (96)	914/2385 (38)	61.3 (7.66); 62 [34, 74]	138.1 (17.4); 137 [87, 234]	84.5 (10.1); 84 [55, 129]	665/2385 (28)	29.2 (4.41) 2385; 28.6 [18.6, 57.3]	5.85 (1.06), 2385	1.29 (0.33), 2382
Multi Ethnic Study of Atherosclerosis (MESA)[9]	6786	6498/6786 (96)	3425/6498 (53)	61.8 (10.13); 62 [44, 84]	126.5 (20.8); 124 [76, 229]	72.2 (10.2); 72 [41, 120]	811/6478 (13)	28.3 (5.46) 6498; 28 [15, 55]	5.03 (0.92), 6475	1.32 (0.38), 6472
Second Manifestations of ARTerial disease (SMART) study[10]	9095	7177/9095 (79)	2512/7177 (35)	55.8 (12.5); 57 [18, 82]	140.4 (20.3); 138 [87, 251]	82.9 (11.8); 81 [48, 210]	1663/7120 (23)	27.1 (4.43) 7171; 26.5 [14.3, 54]	4.93 (1.35), 7139	1.3 (0.39), 7126
Surrogate markers for Micro- and Macrovascular hard endpoints as Innovative diabetes tools (SUMMIT)[11]	375	314/375 (84)	108/314 (34)	66 (8.7); 66 [43, 86]	140.3 (16.0); 139 [105, 189]	78.2 (8.8); 77 [55, 108]	17/314 (5)	29.1 (5.07) 314; 28.5 [18.4, 48.3]	4.47 (1.11), 306	1.42 (0.42), 292
San Diego Population Study[12]	2383	2295/2383 (96)	1516/2295 (66)	58.5 (11.32); 58 [29, 89]	134.2 (20.7); 132 [84, 241]	NR	127/2295 (6)	27 (5.22) 2289; 26.1 [16.2, 53.9]	5.43 (1.05), 2224	1.41 (0.44), 2224
Vietnam Experience Study[13]	4460	4367/4460 (98)	0/4367 (0)	37.8 (2.53); 38 [31, 48]	125.4 (12.2); 125 [85, 200]	85.8 (9.4); 85 [55, 136]	1877/4363 (43)	26.8 (4.42) 4366; 26.2 [15.7, 66.8]	5.49 (1.07), 4367	1.16 (0.32), 4367
Chicago Walking and Leg Circulation Study (WALCS)[14]	442	429/442 (97)	227/429 (53)	69.9 (8.1); 69 [55, 93]	140.8 (19.5); 139 [97, 206]	NR	30/429 (7)	29 (6.14) 426; 28.3 [17, 59.6]	4.63 (1), 393	1.25 (0.47), 393

Note. NR = not reported, BP = blood pressure

Table S2. Distribution of morbidities at baseline for the ABLE-BP dataset

Study	Total participants; N	Total participants included in ABLE-BP analyses; n/N (%)	Hypertension; n/N (%)	Diabetes mellitus; n/N (%)	Ischaemic heart disease; n/N (%)	Cerebrovascular disease; n/N (%)	Any cardiovascular disease; n/N (%)	Renal disease; n/N (%)	Atrial fibrillation / flutter; n/N (%)
Viborg Women Cohort (ViWoCo)[1]	1440	1302/1440 (90)	580/1301 (45)	91/1300 (7)	73/1301 (6)	66/1301 (5)	128/1301 (10)	NR	47/1302 (3.6)
Epidemiology of dementia in Central Africa (EPIDEMCA)[2]	929	697/929 (75)	450/697 (65)	78/695 (11)	NR	9/693 (1)	9/693 (1)	NR	NR
Heinz Nixdorf Recall Study[3]	4736	4391/4736 (93)	2057/4383 (47)	312/4391 (7)	245/4384 (6)	104/4370 (2)	330/4384 (8)	NR	63/4308 (1.5)
Invecchiare in Chianti (InCHIANTI)[4]	1258	1106/1258 (88)	813/1096 (74)	122/1106 (11)	50/1090 (5)	44/1090 (4)	90/1090 (8)	15/208 (7.3)	26/1104 (2.4)
Lifestyle Interventions and Independence for Elders (LIFE) study[5]	1603	1336/1603 (83)	1092/1334 (82)	339/1330 (25)	257/1336 (19)	139/1332 (10)	303/1336 (23)	NR	31/1335 (2.3)
Lahoz 2013 (Fuencarral Health Center)[6]	1103	1092/1103 (99)	532/1092 (49)	158/1092 (14)	5/1092 (0.4)	3/1092 (0)	8/1092 (1)	237/664 (36)	NR
Action for Health in Diabetes (Look AHEAD)[7]	342	321/342 (94)	261/321 (81)	321/321 (100)	34/321 (11)	9/320 (3)	40/321 (12)	16/321 (5)	NR
Improving interMediAte RisK management (MARK) study[8]	2490	2385/2490 (96)	2027/2385 (85)	757/2384 (32)	0/2385 (0)	0/2385 (0)	0/2385 (0)	124/2362 (5.2)	41/2385 (1.7)

Multi Ethnic Study of Atherosclerosis (MESA)[9]	6786	6498/6786 (96)	3259/6498 (50)	845/6475 (13)	0/6498 (0)	0/6498 (0)	0/6498 (0)	137/6473 (2.1)	1/6451 (0.02)
Second Manifestations of ARTerial disease (SMART) study[10]	9095	7177/9095 (79)	5539/7176 (77)	1254/7177 (17)	4449/6579 (68)	1412/7177 (20)	4451/6581 (68)	NR	NR
Surrogate markers for Micro- and Macrovascular hard endpoints as Innovative diabetes tools (SUMMIT)[11]	375	314/375 (84)	197/314 (63)	197/314 (63)	93/314 (30)	32/314 (10)	119/314 (38)	3/314 (1)	0/314 (0)
San Diego Population Study[12]	2383	2295/2383 (96)	760/2292 (33)	129/2294 (6)	125/2295 (5)	56/2295 (2)	166/2295 (7)	NR	NR
Vietnam Experience Study[13]	4460	4367/4460 (98)	2318/4367 (53)	219/4367 (5)	0/4367 (0)	0/4367 (0)	0/4367 (0)	0/4367 (0)	NR
Chicago Walking and Leg Circulation Study (WALCS)[14]	442	429/442 (97)	306/429 (71)	95/429 (22)	143/429 (33)	26/429 (6)	153/429 (36)	NR	NR

Note. NR = not reported. Any cardiovascular disease is defined as individuals with ischaemic heart disease and/or cerebrovascular disease. Total participants, all participants with arm and leg blood pressure readings. Total participants included in ABLE-BP cohort, which excludes those with prior diagnosis of peripheral artery disease, ankle brachial index < 0.90 and leg systolic blood pressure < 70 mmHg.

Table S3 - Study level outcomes and attrition, all participants

Study name	Total participants N	Number of participants with all-cause mortality and time to death; n (%)	Number of participants with CVS cause mortality and time to death; n (%)	Number of participants who had at least one CVS cause event and time to event; n (%)	Duration of follow-up to death or censorship (years); mean (SD), n; median [min, 25th centile, 75th centile, max]	Number of participants with missing mortality status and/or time to mortality; n (%)	Number of participants with missing CVS cause mortality status and/or time to mortality; n (%)	Number of participants with missing CVS event status and/or time to CVS event; n (%)
Viborg Women Cohort (ViWoCo)[1]	1440	32 (2.2)	5 (0.3)	33 (2.3)	3.44 (0.53), 1440; 3.30 [0.41, 2.96, 3.95, 4.22]	0 (0.0)	0 (0.0)	0 (0.0)
Epidemiology of dementia in Central Africa (EPIDEMCA)[2]	1029	110 (10.7)	44 (4.3)	NR	2.01 (0.37), 946; 2.05 [0.11, 1.98, 2.09, 2.69]	83 (8.1)	83 (8.1)	NR
Heinz Nixdorf Recall Study[3]	4814	614 (12.8)	169 (3.5)	588 (12.2)	10.68 (2.36), 4814; 11.48 [0.05, 10.16, 12.30, 14.24]	0 (0.0)	46 (1.0)	0 (0.0)
Invecchiare in Chianti (InCHIANTI)[4]	1453	402 (27.7)	184 (12.7)	NR	7.97 (2.38), 1317; 9.11 [0.10, 7.42, 9.31, 11.42]	136 (9.4)	136 (9.4)	NR
Lifestyle Interventions and Independence for Elders (LIFE) study[5]	1635	133 (8.1)	48 (2.9)	161 (9.8)	3.37 (0.87), 1635; 3.48 [0.00, 3.07, 3.98, 4.52]	0 (0.0)	0 (0.0)	0 (0.0)
Lahoz 2013 (Fuencarral Health Center)[6]	1361	72 (5.3)	13 (1.0)	61 (4.5)	4.26 (0.80), 1301; 4.39 [0.46, 3.78, 4.80, 6.23]	60 (4.4)	61 (4.5)	65 (4.8)
Action for Health in Diabetes (Look AHEAD)[7]	342	30 (8.8)	8 (2.3)	54 (15.8)	10.36 (1.69), 342; 10.90 [1.18, 10.76, 10.94, 11.05]	0 (0.0)	0 (0.0)	0 (0.0)

Improving interMediAte RisK management (MARK) study[8]	2495	26 (1.0)	3 (0.1)	96 (3.8)	3.10 (0.34), 2471; 3.03 [0.07, 3.00, 3.14, 5.22]	24 (1.0)	24 (1.0)	24 (1.0)
Multi Ethnic Study of Atherosclerosis (MESA)[9]	6814	1161 (17.0)	278 (4.1)	911 (13.4)	12.47 (2.50), 6809; 13.20 [0.17, 12.66, 13.74, 14.46]	5 (0.1)	5 (0.1)	5 (0.1)
Second Manifestations of ARTerial disease (SMART) study[10]	11,139	1766 (15.9)	882 (7.9)	1888 (16.9)	7.84 (4.67), 11,139; 7.60 [0.00, 3.96, 11.31, 18.49]	0 (0.0)	0 (0)	0 (0.0)
Surrogate markers for Micro- and Macrovascular hard endpoints as Innovative diabetes tools (SUMMIT)[11]	596	14 (2.3)	1 (0.2)	35 (5.9)	2.93 (0.34), 596; 2.98 [0.57, 2.74, 3.14, 4.08]	0 (0.0)	0 (0.0)	44 (7.4)
San Diego Population Study[12]	2404	473 (19.7)	NR	NR	17.58 (4.36), 2404; 19.21 [0.12, 18.27, 19.91, 20.89]	0 (0.0)	NR	NR
Vietnam Experience Study[13]	4462	250 (5.6)	56 (1.3)	NR	13.13 (2.59), 1555; 13.91 [0.23, 13.54, 14.25, 15.35]	2907 (65.2)	2907 (65.2)	NR
Chicago Walking and Leg Circulation Study (WALCS)[14]	442	45 (10.2)	12 (2.7)	NR	3.98 (1.10), 431; 4.08 [0.16, 4.00, 4.42, 9.74]	11 (2.5)	11 (2.5)	NR

Note. This table presents descriptive data for all reported events, and all durations of follow-up, within the dataset, before censorship at 10-year follow-up. NR = not reported. Any cardiovascular disease (CVS) is defined as individuals with ischaemic heart disease and/or cerebrovascular disease. Total participants, all participants with arm and leg blood pressure readings, which excludes those with prior diagnosis of peripheral artery disease, ankle brachial index < 0.90 and leg systolic blood pressure < 70 mmHg.

Table S4 – Characteristics of included studies

Study name	Period of patient recruitment /Duration of trial	Sample size (n enrolled in study)	Country of origin	Eligibility criteria	Primary outcome measure	Blood pressure measurement methods	Intended maximum duration of follow up	Definition of hypertension	Definition of diabetes	Definition of cardiovascular death and non-fatal cardiovascular event
Viborg Women Cohort (ViWoCo)[1]	October 2011-January 2013	1428	Denmark	Females born in 1936, 1941, 1946 and 1951 living in the Municipal of Viborg, Denmark	Presence of cardiovascular disease and diabetes mellitus	One pair of simultaneous BP readings, using Omron M2 devices, with patients supine, rounded to nearest 2mmHg. Ankle pressures measured synchronously with arms using Doppler probe.	Median follow-up 3.3 years	SBP ≥140 mmHg or DBP ≥90 mmHg	HbA1c ≥ 48 mmol/mol	*Cardiovascular death:* Fatal event as below *Non-fatal event:* MI or ischaemic stroke leading to hospitalisation
Epidemiology of dementia in Central Africa (EPIDEMCA)[2]	November 2011-December 2012	880	Central African Republic/ Republic of Congo	Males and females, aged ≥ 65 years living in areas of Central African Republic and Republic of Congo	Diagnosis of dementia and Alzheimer's disease and associated risk factors	Two sequences of BP measurements recorded using standard mercury sphygmomanometer, as part of ABI protocol with patients supine. BP rounded to nearest 5 mmHg	2-3 years	Self-reported BP lowering treatment; SBP ≥140 mmHg or DBP ≥90 mmHg	Self-reported or blood glucose >126 mg/dL fasting or >200 mg/dL in non-fasting	*Cardiovascular death:* Stroke, MI or other cardiovascular or cerebrovascular diseases – based on interview of relatives during verbal autopsy at follow-up. Non-fatal events: Stroke, MI, other heart disease
Heinz Nixdorf Recall Study[3]	2000-2003	4617	Germany	Males and females, aged 45-74 years, in an unselected urban population from the Ruhr area	Coronary artery calcium as predictor for fatal and non-fatal MI. Secondary endpoints included ABI as a stroke predictor factors	BP measured sequentially using Doppler probe (Logidop, Kranzbuhler, Germany) with patients supine	Mean follow up: 109 months	SBP >140mmHg or DBP >90mmHg	Existing diagnosis or use of anti-diabetic medication	*Cardiovascular death or non-fatal event:* First occurrence of MI based on symptoms, ECG signs, and enzymes, supported by necropsy if fatal

Invecchiare in Chianti (InCHIANTI)[4]	August 1998-March 2000	1091	Italy	Males and females, aged ≥ 65 years, living in Greve and Bagno	Physiological factors influencing walking ability	Single pair of sequential brachial BP readings using standard mercury sphygmomanometer, with patients supine. BP rounded to nearest 5 mmHg. Posterior tibial arteries measured twice with a handheld Doppler stethoscope (Parks model 41-A; Parks Medical Electronics, Inc, Aloha, Ore).	N/S	Self-reported, existing, recorded diagnosis or use of BP lowering medication or SBP ≥140 mmHg or DBP ≥90 mmHg	Self-reported, existing recorded diagnosis, or use of anti-diabetic medication, or fasting glucose >7.0 mmol/L	*Cardiovascular death:* Not defined. *Non-fatal events:* Diagnosis of heart disease, MI or angina, stroke or TIA
Lifestyle Interventions and Independence for Elders (LIFE) study[5]	2010-2011/ 2.6 years	1588	USA	Ambulant community dwelling individuals, aged 70-89 years with a sedentary lifestyle (<20min per week physical activity)	Major mobility disability<br><br>Secondary: Association between ABI and cognitive function	Two pairs of sequential measurements recorded in each arm using handheld Doppler, with patients supine	2 years	Self-reported or measurement	Self-reported	*Cardiovascular fatal or non-fatal events:* MI, angina, stroke or TIA, carotid artery disease, congestive heart failure or PAD requiring hospitalisation, outpatient revascularisation for PAD, ruptured abdominal aortic aneurysm
Lahoz 2013 (Fuencarral Health Center)[6]	2003-2004	1102	Spain	Males and females, aged 60-79 years, with no known PAD	Low ABI and incidence of death due to cardiovascular causes	BP measured sequentially with Doppler 8-MHz probe (Hadeco, Kawasaki, Japan) and calibrated mercury sphygmomanometer with patient supine	Mean follow-up 49.8 months	SBP ≥140 mmHg, DBP ≥90 mmHg or use of BP lowering treatment	Baseline glucose ≥126 mg/dl (>7 mmol/L) on 2 occasions or use of antidiabetic agents	*Cardiovascular death:* Fatal stroke, MI, sudden death without other cause, death after vascular surgery or procedure, death attributed to heart failure, bowel or limb infarction, any other death not categorically attributed to a non-vascular cause *Non-fatal events*: MI, stroke or cardiovascular event

Action for Health in Diabetes (Look AHEAD)[7]	June 2001- March 2004	339	USA	Overweight and obese individuals with type 2 diabetes aged 45-76 years, and had a body mass index, 25 kg/m2, or ≥27 kg/m2 if taking insulin	A composite cardiovascular outcome: cardiovascular death, non-fatal MI, non-fatal stroke, hospitalized angina<br><br>Secondary: Cognitive function	Two pairs of sequential BP measurements recorded in each arm, using continuous wave Doppler with a standard mercury sphygmomanometer, with patients supine	4-5 year follow up	SBP ≥140 mmHg, ≥DBP > 90 mmHg or taking BP lowering medication	Self-reported verified from medical records, current treatment, or fasting glucose of ≥126 mg/dL	*Cardiovascular death:*<br>MI, congestive heart failure, death after cardiovascular intervention, surgery or due to arrhythmia, stroke, presumed cardiovascular death, rapid unexplained cardiovascular death.<br>*Non-fatal events:*<br>Stroke, MI, angina, coronary artery bypass grafting or percutaneous coronary intervention, congestive heart failure, carotid endarterectomy, peripheral arterial bypass or angioplasty
Improving interMediAte RisK management (MARK) study[8]	N/S	2490	Spain	Males and females living in 3 regions of Spain, aged 35-74 years. Free of atherosclerotic disease, with an intermediate cardiovascular risk (10-year coronary risk of 5-15% or vascular death risk of 3-5%) selected at random	Incidence of vascular events	Three pairs of BP measurements in each arm, using an OMRON 705, with patients seated. Legs measured with Vasera device VS-1500® (Fukuda Denshi)	10 years	Patient reported, or use of BP lowering medications or SBP ≥140mmHg or DBP ≥90mmHg	Patient reported, or use of antidiabetic treatment or fasting glucose ≥ 126 mg/dL	*Cardiovascular death:*<br>not defined<br><br>*Non-fatal events*:<br>Stroke or TIA, MI, angina, or revascularisation procedure

Multi Ethnic Study of Atherosclerosis (MESA)[9]	2000-2002	6770	USA	Males and females, aged 45-84 years, free of clinical cardiovascular diagnoses at baseline	Association of subclavian stenosis with markers of cardiovascular disease	Single pair of sequential BP measurements, using hand-held Doppler instrument and 5-mHz probe, with patients supine	N/S	Self-reported history with use of BP lowering medications, or SBP ≥140mmHg or DBP ≥ 90mmHg	Fasting blood glucose ≥126 mg/dl or use of oral hypoglycemic agents or insulin	*Cardiovascular death*: Death due to atherosclerotic coronary heart disease, stroke, other cardiovascular disease. *Non-fatal* events: Stroke, TIA, MI, angina, revascularisation procedure
Second Manifestations of ARTerial disease (SMART) study[10]	January 2002 – February 2014	7600	The Netherlands	Males and females, aged 18-80 years, referred to University Medical Center Utrecht, for treatment of clinically manifest vascular disease or cardiovascular risk factors	3 point MACE (combination of non-fatal myocardial infarction, non-fatal stroke and death from vascular disease), total mortality and vascular mortality	Single pair of sequential BP measurements, using a Vasoguard Doppler probe, with patients supine	Mean follow-up:5.9 years	Blood pressure >140/90 mmHg at baseline or the use of blood pressure lowering medication.	Recorded and self-reported diagnosis, use of blood glucose lowering medication, or fasting glucose >7 mmol/L at recruitment combined with initiation of glucose lowering medication within first year of follow-up. Type 1 diabetes excluded.	*Cardiovascular death:* Death from stroke, MI, congestive heart failure, rupture of abdominal aortic aneurysm or vascular death from other causes<br><br>*Non-fatal events:* Stroke (infarction or haemorrhagic), MI, retinal infarction, heart failure
Surrogate markers for Micro- and Macrovascular hard endpoints as Innovative diabetes tools (SUMMIT)[11]	November 2010 – June 2013	334	England	Adults over 18 with and without diabetes and/or cardiovascular disease		6 pairs of simultaneous BP readings using two Omron 705 devices swapped after 3 readings, with patients supine	N/S	Self-reported history of hypertension	HbA1c ≥ 48 mmol/mol	*Cardiovascular death:* Fatal MI

San Diego Population Study[12]	1994-1998	2388	USA	Males and females, aged 29-91 years, attending a clinic for assessment of PAD and venous disease	Prevalence of PAD	Two pairs of BP measurements, using a continuous-wave Doppler ultrasound, with patients supine	N/S	SBP ≥140 mmHg or DBP ≥ 90 mmHg or use of BP lowering medications	Self-reported or use of antidiabetic medications	*Cardiovascular death:* not defined *Non-fatal events:* MI, stroke, angina, coronary angioplasty or bypass graft, or carotid endarterectomy
Vietnam Experience Study[13]	1986	4394	USA	Male US army veterans who participated in the Vietnam war	Inter-arm differences, all-cause and cardiovascular mortality	Two pairs of sequential brachial and ankle measurements using a Model 1010-LA Dual Frequency BiDirectional Doppler (Parks Medical Electronics, Beaverton, Oregon, USA) doppler probe with participant supine	15 years	SBP ≥140 mmHg, DBP ≥90 mmHg or use of BP lowering medication	Fasting plasma glucose ≥ 7.0 mmol/l and/or use of medication for diabetes	*Cardiovascular death:* Death due to major cardiovascular disease.
Chicago Walking and Leg Circulation Study (WALCS)[14]	1998-2000	440	USA	Patients without lower extremity peripheral artery disease who were recruited for the non-PAD comparator group.	Subclavian stenosis as a marker for total and cardiovascular disease mortality	Two sequences of BP readings recorded using a 12-cm pneumatic cuff and a hand held Doppler probe (Nicolet Vascular Pocket Dop II, Golden, Colo) with patient supine	Mean follow-up was 4.8 years.	Patient history or use of BP lowering therapy	Patient history or use of oral antidiabetic drugs and/or insulin	*Cardiovascular death*: Any fatal cardiovascular cause. *Non-fatal events*: MI, stroke, TIA, coronary or peripheral revascularisation, congestive heart failure, PAD, angina

BP = BP, DBP = diastolic BP, IHD = ischaemic heart disease, MI = myocardial infarction, N/S = not stated, PAD = peripheral arterial disease, SBP = systolic BP, TIA = transient ischaemic attack, ECG = electrocardiogram

Table S5. Ankle-arm systolic blood pressure prediction model using observed and imputed data (derivation cohort)

	Coefficient	Lower bound of 95% CI	Upper bound of 95% CI	P value
Higher-reading ankle SBP (mmHg)	0.60	0.59	0.60	<0.001
Age (years)	0.19	0.17	0.20	<0.001
Female	4.30	3.95	4.64	<0.001
Smoker	2.05	1.69	2.42	<0.001
BMI (Kg/m^2)	0.02	-0.01	0.05	0.15
Total cholesterol (mmol/L)	0.74	0.60	0.89	<0.001
Hypertension	6.09	5.75	6.43	<0.001
Diabetes mellitus	0.56	0.14	0.98	0.009
Cerebrovascular disease	1.47	0.79	2.16	<0.001
Ischaemic heart disease	-2.87	-3.50	-2.24	<0.001
Ethnicity				
- African American	2.45	1.90	3.00	
- Hispanic American	-0.25	-0.92	0.43	[1]<0.001
- Other	0.04	-0.42	0.49	
Constant	23.67	20.69	26.64	<0.001

Note. [1]Global p-value. Confidence interval, CI; Systolic blood pressure, SBP; Body mass index, BMI.

Table S6 – Comparison of models' classification of estimated systolic blood pressure to observed systolic blood pressure at A) 140 mmHg and B) 160 mmHg thresholds

**A**	**ABLE-BP model**		**Arithmetic models**		**Totals**
Observed arm SBP category	*Estimated arm SBP category*		*Estimated arm SBP category*		
	<140 mmHg	≥140 mmHg	<140 mmHg	≥140 mmHg	
**<140 mmHg**	14,694	3,523	14,432	3,785	18,217
	45.14%	9.76%	44.34%	11.63%	
**≥140 mmHg**	3,177	11,157	3,545	10,789	14,334
	10.82%	34.28%	10.89%	33.14%	
					32,551
**N misclassified**		6,700		7,330	
**% misclassified**		20.6%		22.5%	

**B**	**ABLE-BP model**		**Arithmetic models**		**Totals**
Observed arm SBP category	*Estimated arm SBP category*		*Estimated arm SBP category*		
	<160 mmHg	≥160 mmHg	<160 mmHg	≥160 mmHg	
**<160 mmHg**	26,351	1,136	25,297	2,190	27,487
	80.95%	7.95%	77.71%	6.73%	
**≥160 mmHg**	2,589	2,475	2,160	2,904	5,064
	3.49%	7.60%	6.64%	8.92%	
					32,551
**N misclassified**		3,725		4,350	
**% misclassified**		11.4%		13.4%	

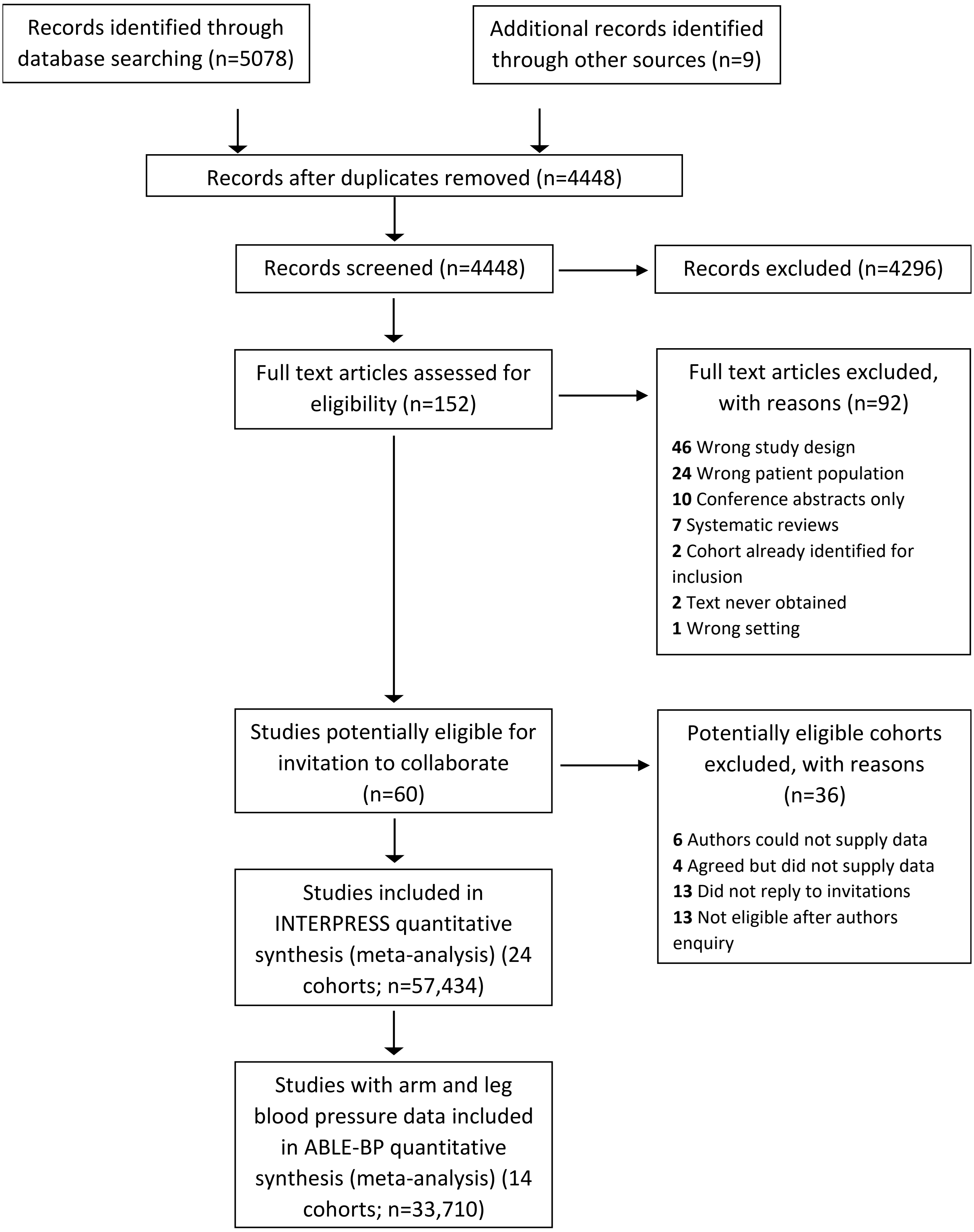

Figure S1. PRISMA flow chart for literature search

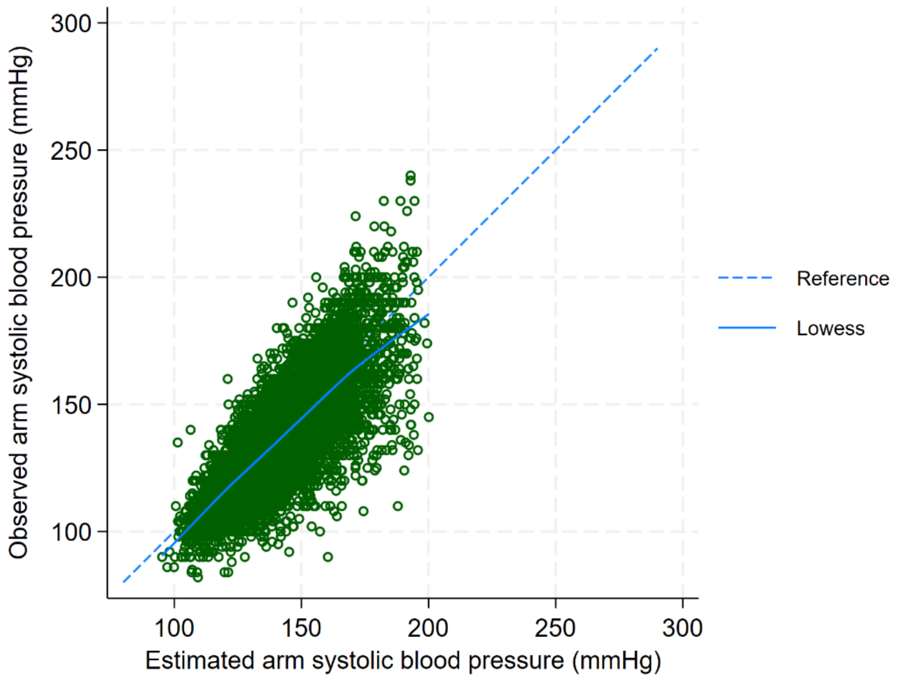

a) Derivation dataset

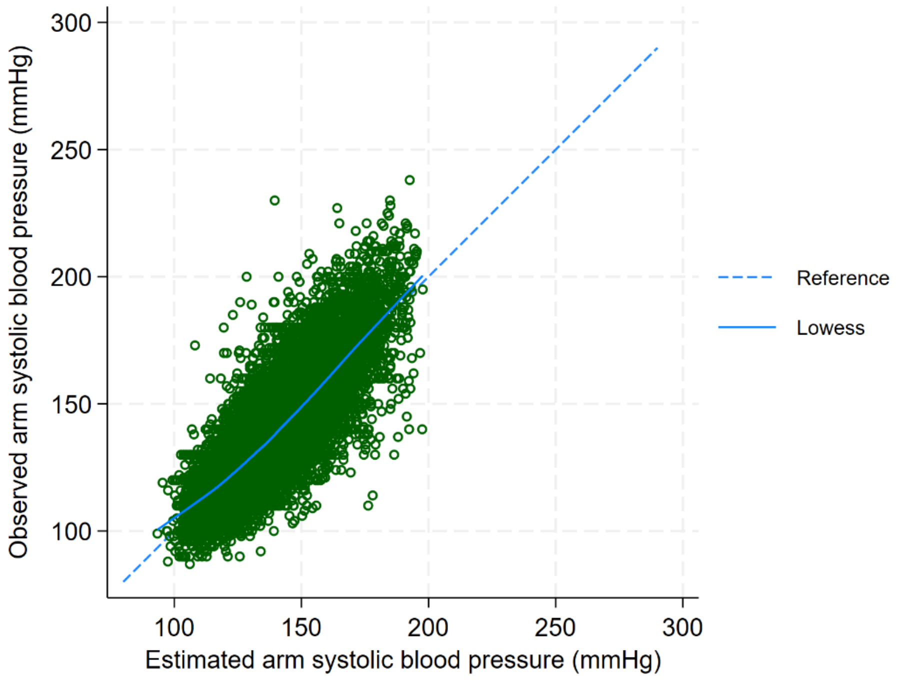

b) Validation dataset

Figure S2 – Calibration plots for observed arm systolic blood pressures against model derived expected arm systolic blood pressures for a) derivation and b) validation datasets

References to included studies

1. Dahl M, Frost L, Søgaard R, et al. A population-based screening study for cardiovascular diseases and diabetes in Danish postmenopausal women: acceptability and prevalence. *BMC cardiovascular disorders* 2018;18(1):20. doi: 10.1186/s12872-018-0758-8
2. Guerchet M, Mbelesso P, Ndamba-Bandzouzi B, et al. Epidemiology of dementia in Central Africa (EPIDEMCA): protocol for a multicentre population-based study in rural and urban areas of the Central African Republic and the Republic of Congo. *SpringerPlus* 2014;3:338. doi: 10.1186/2193-1801-3-338 [published Online First: 2014/07/22]
3. Erbel R, Mohlenkamp S, Moebus S, et al. Coronary risk stratification, discrimination, and reclassification improvement based on quantification of subclinical coronary atherosclerosis: the Heinz Nixdorf Recall study. *J Am Coll Cardiol* 2010;56(17):1397-406. doi: 10.1016/j.jacc.2010.06.030 [published Online First: 2010/10/16]
4. Clark CE, Thomas D, Llewellyn DJ, et al. Systolic inter-arm blood pressure difference and risk of cognitive decline in older people: a cohort study. *British Journal of General Practice* 2020:bjgp20X709589. doi: 10.3399/bjgp20X709589
5. Espeland MA, Newman AB, Sink K, et al. Associations Between Ankle-Brachial Index and Cognitive Function: Results From the Lifestyle Interventions and Independence for Elders Trial. *J Am Med Dir Assoc* 2015;16(8):682-9. doi: 10.1016/j.jamda.2015.03.010 [published Online First: 2015/04/15]
6. Lahoz C, Barrionuevo M, Garcia-Fernandez T, et al. Cardiovascular morbidity-mortality associated to ankle-brachial index in the general population. [Spanish]. *Revista Clinica Espanola* 2014;214(1):1-7.
7. Espeland MA, Beavers KM, Gibbs BB, et al. Ankle-brachial index and inter-artery blood pressure differences as predictors of cognitive function in overweight and obese older adults with diabetes: Results from the Action for Health in Diabetes movement and memory study. *International Journal of Geriatric Psychiatry* 2015;30(10):999-1007.
8. Martí R, Parramon D, García-Ortiz L, et al. Improving interMediAte risk management. MARK study. *BMC cardiovascular disorders* 2011;11:61. doi: 10.1186/1471-2261-11-61 [published Online First: 2011/10/14]
9. Bild DE, Bluemke DA, Burke GL, et al. Multi-Ethnic Study of Atherosclerosis: Objectives and Design. *American Journal of Epidemiology* 2002;156(9):871-81.
10. Kranenburg G, Spiering W, de Jong PA, et al. Inter-arm systolic blood pressure differences, relations with future vascular events and mortality in patients with and without manifest vascular disease. *International journal of cardiology* 2017;244:271-76. doi: 10.1016/j.ijcard.2017.06.044
11. Clark CE, Casanova F, Gooding K, et al. Inter-arm blood pressure difference and arterial stiffness. *Journal of Hypertension* 2014;32(eSuppl A):e30.
12. Wassel CL, Loomba R, Ix JH, et al. Family History of Peripheral Artery Disease is associated with Prevalence and Severity of Peripheral Artery Disease: The San Diego Population Study (SDPS). *Journal of the American College of Cardiology* 2011;58(13):1386-92. doi: 10.1016/j.jacc.2011.06.023
13. White J, Mortensen LH, Kivimaki M, et al. Interarm differences in systolic blood pressure and mortality among US army veterans: aetiological associations and risk prediction in the Vietnam experience study. *EurJPrevCardiol* 2014;21(11):1394-400.
14. McDermott MM, Greenland P, Liu K, et al. Leg symptoms in peripheral arterial disease: associated clinical characteristics and functional impairment. *JAMA* 2001;286(13):1599-606.
