# Supplementary material for: Arm Based on LEg blood pressures (ABLE-BP): can systolic ankle blood pressure measurements predict systolic arm blood pressure? An individual participant data meta-analysis from the INTERPRESS-IPD Collaboration
Source: BMJ Open. 2025 Jun 11;15(6):e094389. doi: 10.1136/bmjopen-2024-094389 (PMC12161303; doi:10.1136/bmjopen-2024-094389)
Supplement: Supplementary file 2 [file bmjopen-2024-094389.reviewer_comments.pdf]

PEER REVIEW HISTORY

BMJ Open publishes all reviews undertaken for accepted manuscripts. Reviewers are asked to complete a checklist review form and are provided with free text boxes to elaborate on their assessment. These free text comments are reproduced below.

This paper was submitted to another journal from BMJ but declined for publication following peer review. The authors addressed the reviewers' comments and submitted the revised paper to BMJ Open. The paper was subsequently accepted for publication at BMJ Open.

ARTICLE DETAILS

Title (Provisional)

Arm Based on LEg blood pressures (ABLE-BP): Can systolic ankle blood pressure measurements predict systolic arm blood pressure? An individual participant data meta-analysis from the INTERPRESS-IPD Collaboration

Authors

McDonagh, Sinead TJ; Warren, Fiona C; Sheppard, James Peter; Boddy, Kate; Farmer, Leon; Shore, Helen; Williams, Phil; Lewis, Philip S; Fordham, A Jayne; Martin, Una; Aboyans, Victor; Clark, Christopher Elles; authors, INTERPRESS-IPD Collaboration

---

VERSION 1 – REVIEW

**Reviewer**	**1**
**Name**	**Reviewer 1**
**Affiliation**	
**Date**	**21-Jun-2024**
**COI**	**No competing interest**

Comments not published pending reviewer's consent

**Reviewer**	**2**
**Name**	**Auer, Johann**
**Affiliation**	**General Hospital Braunau, Cardiology and Intensive Care**
**Date**	**21-Jun-2024**
**COI**	**No competing interest**

This study investigated the association between arm and ankle systolic BP and to developed and validated a model that accurately estimates arm systolic blood pressure from ankle systolic blood

pressure measurements and readily available participant characteristics.   Additionally, a calculator based on this model has been created to facilitate interpretation of blood pressure values when only ankle pressures are available. Finally, the authors assessed associations between ankle BP, cardiovascular disease, and mortality.
The issue is worth to be investigated. The paper is well written. However, there are some major points that have to be addressed:

The authors should add information regarding differences in BP measurement protocols between studies. Pooling of a heterogenous population and different analytical techniques may limit the value of this findings.
Significant residual heterogeneity points in a similar direction and may underscore substantial limitation in the findings from this metaanalysis.

Was BP measurement in all four limbs performed simultaneously or sequentially (or either/or) in all study subjects?

The authors state that individual participants were excluded if they had PAD, an ABI <0.90, or lower reading ankle SBP <70 mmHg or >250mmHg since such readings are either diagnostic or highly indicative of PAD. Rhis definition suggests a large „grey zone" and may indicate that a lot of unrecognized PAD patients have been included in this study. This could have introduced error and may invalidate results.

Ankle BP was neither associated with all-cause nor cardiovascular mortality. Are these findings unexpected tot he authors. Are this findings plausible?

Why did the authors not apply a random effects model?

**Reviewer**	**3**
**Name**	**Reviewer 3**
**Affiliation**	
**Date**	**21-Jun-2024**
**COI**	**No competing interest**

Comments not published pending reviewer's consent

VERSION 1 - AUTHOR RESPONSE

Reviewer: 2

Dr. Johann Auer, General Hospital Braunau

This study investigated the association between arm and ankle systolic BP and to developed and validated a model that accurately estimates arm systolic blood pressure from ankle systolic blood pressure measurements and readily available participant characteristics. Additionally, a calculator based on this model has been created to facilitate interpretation of blood pressure values when only ankle pressures are available. Finally, the authors assessed associations between ankle BP, cardiovascular disease, and mortality.
The issue is worth to be investigated. The paper is well written. However, there are some major points that have to be addressed:

1. The authors should add information regarding differences in BP measurement protocols

between studies. Pooling of a heterogenous population and different analytical techniques may limit the value of this findings.

Thank you – all measures were obtained by the individual studies using sequential ankle-brachial index protocols with doppler probes. A line on BP measurement has been added to methods and reference is made to our previous INTERPRESS paper which summarised this data.

2. Significant residual heterogeneity points in a similar direction and may underscore substantial limitation in the findings from this metaanalysis.

With the exception of the ViWoCo and SUMMIT cohorts, the mean ankle-arm difference across studies lies between 10.28 and 17.32, which are comparable in terms of order of magnitude (Figure 2). Statistical heterogeneity is high due to the narrow confidence intervals associated with study estimates.

3. Was BP measurement in all four limbs performed simultaneously or sequentially (or either/or) in all study subjects?

Sequential in all cases – this has been addressed in the text in responding to point 1, above

4. The authors state that individual participants were excluded if they had PAD, an ABI <0.90, or lower reading ankle SBP <70 mmHg or >250mmHg since such readings are either diagnostic or highly indicative of PAD. Rhis definition suggests a large „grey zone“ and may indicate that a lot of unrecognized PAD patients have been included in this study. This could have introduced error and may invalidate results.

Thank you for raising this point. It is, of course, impossible to state that all participants were free from undiagnosed PAD. Since members of the "grey zone" referred to met no non-invasive diagnostic criteria for suspecting or diagnosing PAD, there were no objective grounds to exclude them. Such people, with occult disease, will naturally form part of any cohort representative of the wider community. A line has been added to strengths and weaknesses to acknowledge this point.

5. Ankle BP was neither associated with all-cause nor cardiovascular mortality. Are these findings unexpected tot he authors. Are this findings plausible?

Thank you. We consider that our exclusion of both low and high extremes of ankle SBP is likely to have weakened the association, previously demonstrated, of ankle SBP with mortality. we have acknowledged this association and addressed it in two sentences in the discussion.

6. Why did the authors not apply a random effects model?
The random effects model did not converge, therefore, as planned in our published protocol, we proceeded to fixed effect modelling. This is stated in the methods.

VERSION 2 - REVIEW

Reviewer 2

Name Auer, Johann

Affiliation General Hospital Braunau, Cardiology and Intensive Care

Date 20-Oct-2024

COI None

.

Reviewer 4

Name Lakhal, Karim

Affiliation Centre Hospitaler Universitaire de NANTES, Anesthésie et réanimation chirurgicale, Hôpital Guillaume et René Laënnec

Date 25-Nov-2024

COI None

I have read with interest the study by McDonagh et al. entitled « Arm Based on LEg blood pressures (ABLE-BP): Can systolic ankle blood pressure measurements predict systolic arm blood pressure? An individual participant data metaanalysis from the INTERPRESS-IPD Collaboration ».

In this IPD meta-analysis of BP collected in more than 33,700 patients (14 studies), the authors

- found that ankle systolic BP was, on average, 12.0 mmHg (95% confidence interval 8.8 to 15.2) higher than the arm.

- developed and validated a model that derives arm systolic BP from the ankle systolic BP for the detection of arterial hypertension. This model has honorable performance (AUROC curves, sensitivities and specificities >0.82, 0.80 and 0.82).

- assessed the relationship between ankle BP, on the one hand, and mortality and cardiovascular events.

The topic is interesting, the study size is impressive, the paper is well written, and there are no ethical concerns. Below are some specific comments.

- The device used for BP measurement appears to be overlooked in both the analyses and the paper as a whole. However, BP readings can vary depending on the technique

(oscillometry vs. auscultation), the manufacturer, and even the specific model or version of the device.

- Please specify in the abstract that measurements were taken with patients in the supine position, as ankle BP measurements are known to be influenced by patient positioning (‘Noninvasive Arterial Pressure Measurements at the Lower Leg: Evaluation of the Influence of Patient Position (Semi-Recumbent or Horizontal)-A prospective study.’, Lakhal K et al, Chest 2024, PMID: 39236997).

- The abstract mentions that the ‘higher-reading ankle systolic BP’ was used for the analysis, while the Methods section states: ‘We defined the difference between arm and ankle BP as the lower-reading posterior tibial artery SBP minus the higher-reading arm SBP.’ I may have misunderstood the subtleties regarding the choice of higher vs. lower systolic BP at the ankle. Could you please clarify this? Additionally, could you indicate whether a significant difference between ankles (and between arms) was observed?

- Please clarify throughout the manuscript that the second objective (predicting arm BP from ankle BP) specifically refers to the detection of hypertension, as the detection of hypotension was not studied.

- Comparing the model's performance with existing formulae could be included as an additional objective of the study. The same applies to evaluating the association between the estimated arm BP (derived from ankle BP) and cardiovascular outcomes.

- Comparison of the model's performance with existing formulae (Table S5): the improvement appears very limited (misclassification rate: 20.6% vs. 22.5%). Additionally, no statistical comparison is provided. Therefore, the statement in the abstract, ‘Model performance was superior to all existing fixed formulae,’ may not be fully supported by the data. Same for ‘We developed and validated a model to estimate arm BP from ankle BP which demonstrated greater accuracy than current arithmetic methods of conversion’ (Discussion).

- In my opinion, an AUC-ROC of 0.82 does not justify being described as ‘excellent,’ as claimed by the authors. Terms like ‘honorable’ or ‘fair’ seem more appropriate.

- The exclusion of participants with diagnosed or suspected periph. art. disease from the analyses should be mentioned in the highlights box ‘Strengths and limitations of this study’ and in the abstract.

- As the authors are well aware, in acute care settings, factors such as IV lines, surgeries, fractures, or burns often necessitate placing the cuff on the lower leg. However, it appears that patients from acute care settings were not included in the present study. Therefore, it should be emphasized that the findings should not be extrapolated to this population.

- I am unable to provide a reliable assessment of the statistical methods presented.

The concept of ‘AUC at cut point’ is unclear to me and possibly to many readers. It may deserve some clarification (in the Legend of Table 4, for instance). Same for ‘Estimated optimal

Cutpoint’.

Could Sensitivity and Specificity values be accompanied by their confidence interval (Table 4, Results, and abstract)?

Fig 2.: Confidence intervals are not provided.

**Reviewer**	**5**
**Name**	**Dugar, Siddharth**
**Affiliation**	**Cleveland Clinic, Respiratory Institute**
**Date**	**09-Dec-2024**
**COI**	**None**

The author have satisfactorily responded to all concerns I had while reading the paper and the other reviewers' comments from BMJ. The methodology is apt.

**Reviewer**	**6**
**Name**	**Huang, Li-Ching**
**Affiliation**	**Vanderbilt University, Biostatistics**
**Date**	**03-Jan-2025**
**COI**	**None**

1. Please remove the study results from the Introduction section.

2. Table 1 shows the 16 participants did not have systolic blood pressure. These participants should be excluded from the study due to have no primary outcome data.

3. Table 1 shows there is no missing value in age, sex, smoking status, hypertension, diabetes mellitus, cardiovascular disease and ethnicity. The N=33710. Please explain why the multiple imputation should be applied in regression analysis.

4. Table 1 should include all the variables which were in multivariable regression models.

5. Table 2, the outcome is ankle-arm SBP difference. Please explain why the higher-reading arm SBP was included in the model.

6. It looks like the authors have patient-level data for this study. It is unclear to me why the authors use one-stage and two-stage meta-analysis to perform the analysis. Why not just used the regression analysis?

VERSION 2 - AUTHOR RESPONSE

Reviewer comments

Reviewer: 2

Dr. Johann Auer, General Hospital Braunau

Comments to the Author:

This section was blank

Reviewer: 4

Dr. Karim Lakhal, Centre Hospitaler Universitaire de NANTES

Comments to the Author:

I have read with interest the study by McDonagh et al. entitled « Arm Based on LEg blood pressures (ABLE-BP): Can systolic ankle blood pressure measurements predict systolic arm blood pressure? An individual participant data metaanalysis from the INTERPRESS-IPD Collaboration ».

In this IPD meta-analysis of BP collected in more than 33,700 patients (14 studies), the authors

- found that ankle systolic BP was, on average, 12.0 mmHg (95% confidence interval 8.8 to 15.2) higher than the arm.
- developed and validated a model that derives arm systolic BP from the ankle systolic BP for the detection of arterial hypertension. This model has honorable performance (AUROC curves, sensitivities and specificities >0.82, 0.80 and 0.82).
- assessed the relationship between ankle BP, on the one hand, and mortality and cardiovascular events.

The topic is interesting, the study size is impressive, the paper is well written, and there are no ethical concerns. Below are some specific comments.

- The device used for BP measurement appears to be overlooked in both the analyses and the paper as a whole. However, BP readings can vary depending on the technique (oscillometry vs. auscultation), the manufacturer, and even the specific model or version of the device.

Thank you for your comment. In our original published ABLE-BP protocol paper, we present the BP device used by each study and this is outlined in detail in Table 1 in that paper (which is referenced throughout the current paper). We have now added this table of included study characteristics to the supplement – please see Table S4 and added to the results section – 2nd sentence. Subgroup analyses by device was not considered feasible since Doppler probes were predominantly used, according to ABI measurement protocols, for ankle pressure measurements throughout our included studies. A caution against other e.g. oscillometric ankle pressure measurements has been added to strengths and weaknesses.

- Please specify in the abstract that measurements were taken with patients in the supine position, as ankle BP measurements are known to be influenced by patient positioning ('Noninvasive Arterial Pressure Measurements at the Lower Leg: Evaluation of the Influence of Patient Position (Semi-Recumbent or Horizontal)-A prospective study.', Lakhal K et al, Chest 2024, PMID: 39236997).

Thank you for your comment. We have now added that measurements were made in the supine position to the abstract. Please also see reference to this in the main methods and discussion sections as well as Table S4. Importance of supine position is now cited in strengths and limitations

- The abstract mentions that the 'higher-reading ankle systolic BP' was used for the analysis, while the Methods section states: 'We defined the difference between arm and ankle BP as the lower-reading posterior tibial artery SBP minus the higher-reading arm SBP.' I may have misunderstood the subtleties regarding the choice of higher vs. lower systolic BP at the ankle. Could you please clarify this?

Thank you for the comment. We defined the difference between arm and ankle BP as the lower-reading posterior tibial artery SBP minus the higher-reading arm SBP using the first set of four-limb BP readings – this was predefined and stated in the protocol paper and used to support us in meeting aim 1 of this paper – to describe the relationship of arm and leg SBP.

We then ran models using either the higher or the lower reading ankles for 'arm based on ankle BP prediction' models (to meet aim 2) and 'time-to-event models' (to meet aim 3). These utilised either the higher-reading or the lower-reading ankle BP in separate models. The difference between arm and leg BP as defined above was not part of these models. This process is explained in the methods and results sections in detail (results highlighted below).

The model utilising higher-reading ankle SBP demonstrated better model fit for estimating higher arm SBP than that using lower-reading ankle SBP (AICs: 163292 vs 163962 respectively) - hence described in the abstract as a main finding.

For participants without pre-existing cardiovascular disease, neither lower- nor higher-reading ankle SBP were associated, after adjustment for other predictors, with either all-cause mortality (adjusted HRs 1.00 (95% CI 1.00 to 1.00; P=0.682) & 1.00 (1.00 to 1.00; P=0.796) respectively) or cardiovascular mortality (HRs 1.00 (1.00 to 1.01;P=0.840) & 1.00 (1.00 to 1.01; P=0.297) respectively).

Lower-reading ankle SBP was significantly associated with fatal and non-fatal cardiovascular events (HR 1.005, (1.002 to 1.007; P<0.001)).

Additionally, could you indicate whether a significant difference between ankles (and between arms) was observed?
Thank you for this comment. This study did not determine inter-arm or inter-ankle differences, these analyses were not proposed in our published protocol so are not pursued in this manuscript. We do however, report the ankle-arm difference, as shown in the results section and in figure 1.

We have previously published, in depth, concerning the prevalence and prognostic implications of an inter-arm SBP difference, using the larger dataset on which the current manuscript was based (Please see Clark CE, Warren FC, Boddy K, et al. Associations Between Systolic Interarm Differences in Blood Pressure and Cardiovascular Disease Outcomes and Mortality. Hypertension 2021;77:650-61. doi: 10.1161/HYPERTENSIONAHA.120.15997 [published Online First: 21/12/2020] – cited in the current manuscript).

Inter-ankle difference has been far less researched than inter-arm difference (see: Four-limb blood pressure measurement: a research tool looking for a clinical use. C. E. Clark. *Hypertension* 2013 Vol. 61 Issue 1146 Pages 1147). It was not an aim of the current analyses and remains a future, separate, study plan.

Consequently, we do not discuss inter-arm or inter-ankle differences in the current manuscript.

- Please clarify throughout the manuscript that the second objective (predicting arm BP from ankle BP) specifically refers to the detection of hypertension, as the detection of hypotension was not studied.
Thank you for your comment. The aim of this study was to derive and validate a model to estimate arm SBP based on ankle SBP, irrespective of hypertension status. We have then demonstrated the potential practical application of this model by applying it to commonly used hypertension diagnostic thresholds. We have clarified this in the methods and explicitly stated that it was outside the scope of this study to apply our model to commonly used hypotension or postural hypotension thresholds.

- Comparing the model's performance with existing formulae could be included as an additional objective of the study. The same applies to evaluating the association between the estimated arm BP (derived from ankle BP) and cardiovascular outcomes.
Thank you for the comment. Our aims are in line with our published protocol. Comparison with existing arithmetic models was a post-hoc analysis – not a prespecified objective therefore we include these comparisons for illustrative purposes rather than a claimed objective. We can modify this approach should the editors wish.

We believe the 3rd aim of our study 'investigate the associations between ankle BP cardiovascular disease and mortality', answers your query re. cardiovascular outcomes.

- Comparison of the model's performance with existing formulae (Table S5): the improvement appears very limited (misclassification rate: 20.6% vs. 22.5%). Additionally, no statistical comparison is provided. Therefore, the statement in the abstract, 'Model performance was superior to all existing fixed formulae,' may not be fully supported by the data. Same for 'We developed and validated a model to estimate arm BP from ankle BP which demonstrated greater accuracy than current arithmetic methods of conversion' (Discussion).
Thank you for your comment. Table S6 (previously Table S5) demonstrates that our model is superior to existing methods and we demonstrate this at commonly used hypertension thresholds of 140 and 160 mmHg systolic blood pressure. Significance (P<0.0001) is reported in the corresponding text (results/ Arm blood pressure estimated from ankle blood pressure – final paragraph).
Using a two-sample test of proportions, the difference in proportions (ABLE-BP algorithm minus arithmetic model) is –0.019 (95% confidence interval –0.26 to –0.013, p-value< 0.0001). Although a 2% misclassification may seem small, we argue that in the clinical context, use of the ABLE-BP algorithm in preference to an arithmetic model represents an improvement in diagnostic classification of hypertension for a substantive number of patients. Hypertension is common. The NHS Health Check Programme diagnoses 38,000 new cases of hypertension annually in England alone (Robson J, Dostal I, Sheikh A, *et al*. The NHS Health Check in England: an evaluation of the first 4 years. *BMJ Open* 2016; 6(1): e008840. DOI: https://doi.org/10.1136/bmjopen-2015-008840).
Therefore, a 2% reduction in misclassification potentially represents 760 missed diagnoses per year in England, or many thousands globally. We now state this in the discussion.

- In my opinion, an AUC-ROC of 0.82 does not justify being described as 'excellent,' as claimed by the authors. Terms like 'honorable' or 'fair' seem more appropriate.
Thank you for the comment. We politely disagree with this comment – an AUROC >0.8 is commonly reported as 'Excellent'. We now add a reference to support this convention (see Methods/statistical analysis/estimating arm blood pressure…). Ref: "Receiver Operating Characteristic Curve in Diagnostic Test Assessment": https://www.sciencedirect.com/science/article/pii/S1556086415306043

- The exclusion of participants with diagnosed or suspected periph. art. disease from the analyses should be mentioned in the highlights box 'Strengths and limitations of this study' and in the abstract.
Thank you for your comment. We already include the following statement re. exclusion of individuals with suspected or diagnosed PAD in the 'strengths and limitations of this study' section:
'Finally, we necessarily excluded participants with diagnosed or suspected PAD from our analyses. PAD may significantly and unpredictably alter the relationship between ankle and arm SBP, making it inappropriate to apply our models to people with known or suspected PAD. Participants not meeting diagnostic criteria for PAD will undoubtedly have been represented in the cohorts studied, as would be expected for any sample representative of a wider community. '
We have added a sentence to the abstract re. PAD exclusion criterion.

- As the authors are well aware, in acute care settings, factors such as IV lines, surgeries, fractures, or burns often necessitate placing the cuff on the lower leg. However, it appears that patients from acute care settings were not included in the present study. Therefore, it should be emphasized that the findings should not be extrapolated to this population.
Thank you for your comment. We have added a sentence to the strengths and limitations section: 'It is important to note that data from acute care settings were not included in these analyses and therefore findings cannot be extrapolated to this population.' We already include a statement of intended use – not for emergency or acute care settings – on our able-bp calculator. However, in light of your comments, we are pleased to clarify that statement.

- I am unable to provide a reliable assessment of the statistical methods presented.
The concept of 'AUC at cut point' is unclear to me and possibly to many readers. It may deserve some clarification (in the Legend of Table 4, for instance). Same for 'Estimated optimal Cutpoint'.

Thank you for the comment. We have edited the text and Table 4 to improve clarity of our terminology and presentation of results. We hope this is now acceptable.

Could Sensitivity and Specificity values be accompanied by their confidence interval (Table 4, Results, and abstract)?
Fig 2.: Confidence intervals are not provided.
Thank you for the comment. We have revised Table 4 to include 95% CI for sensitivity, specificity, AUC, for validation and derivation cohorts separately. We could also add to the results and abstract, but prefer not to further stress the abstract word limit. We can review this should the editors consider it important.

Reviewer: 5
Dr. Siddharth Dugar, Cleveland Clinic
Comments to the Author:
The author have satisfactorily responded to all concerns I had while reading the paper and the other reviewers' comments from BMJ. The methodology is apt.
We thank the reviewer for the comment.

Reviewer: 6
Dr. Li-Ching Huang, Vanderbilt University
Comments to the Author:
1. Please remove the study results from the Introduction section.
Thank you for the comment. We describe the previous study-level results in the introduction, not the results for the current IPD. We have clarified and added the word 'previous' to the introduction – we hope this is sufficient.

2. Table 1 shows the 16 participants did not have systolic blood pressure. These participants should be excluded from the study due to have no primary outcome data.
Thank you for your comment. Table 1 describes the eligible cohort at baseline. We restricted analyses to complete case data. We have highlighted this in the methods section. We believe no further action is needed.

3. Table 1 shows there is no missing value in age, sex, smoking status, hypertension, diabetes mellitus, cardiovascular disease and ethnicity. The N=33710. Please explain why the multiple imputation should be applied in regression analysis.
Thank you for the comment. Missing data at baseline can be inferred from Table 1 (for variables including SBP, BMI, total cholesterol, HDL, IHD and CVD etc.). The imputation analysis was undertaken for missing data at baseline and models rerun as sensitivity analyses only.

4. Table 1 should include all the variables which were in multivariable regression models.
Thank you for the comment. We have added ischaemic heart disease, cerebrovascular disease and lower leg SBP to Table 1.

5. Table 2, the outcome is ankle-arm SBP difference. Please explain why the higher-reading arm SBP was included in the model.
Thank you for the comment. As correctly pointed out by the reviewer, the outcome in this regression model is the difference in blood pressure i.e. ankle BP minus arm BP. Higher arm BP is included in the model so that any association between higher arm BP and ankle-arm difference can be estimated.

6. It looks like the authors have patient-level data for this study. It is unclear to me why the authors use one-stage and two-stage meta-analysis to perform the analysis. Why not just used the regression analysis?
As the reviewer has commented, we have individual patient data for these primary studies. We also used 2-stage meta-analysis models to complement our 1-stage regression models, by producing forest plots, and allowing assessment of between study heterogeneity using the I^2 statistic.

Reviewer: 2
If you have selected 'Yes' above, please provide details of any competing interests.: .

Reviewer: 4
If you have selected 'Yes' above, please provide details of any competing interests.: Not applicable

Reviewer: 5
If you have selected 'Yes' above, please provide details of any competing interests.: None

Reviewer: 6
If you have selected 'Yes' above, please provide details of any competing interests.: Not applicable

---

VERSION 3- REVIEW

**Reviewer**	**4**
**Name**	**Lakhal, Karim**
**Affiliation**	**Centre Hospitaler Universitaire de NANTES, Anesthésie et réanimation chirurgicale, Hôpital Guillaume et René Laënnec**
**Date**	**02-Apr-2025**
**COI**	

The authors have satisfactorily responded to my comments & questions.
